# Preliminary Study of MR Diffusion Tensor Imaging of the Liver for the Diagnosis of Hepatocellular Carcinoma

**DOI:** 10.1371/journal.pone.0135568

**Published:** 2015-08-28

**Authors:** Xinghui Li, Qi Liang, Ling Zhuang, Xiaoming Zhang, Tianwu Chen, Liangjun Li, Jun Liu, Horea Calimente, Yinan Wei, Jiani Hu

**Affiliations:** 1 Sichuan Key Laboratory of Medical Imaging, Department of Radiology, Affiliated Hospital of North Sichuan Medical College, Nanchong, 637000, China; 2 Department of Laboratory, Affiliated Hospital of North Sichuan Medical College, Nanchong, 637000, China; 3 Department of Radiation Oncology, Wayne State University, Detroit, 48201, MI, United States of America; 4 Department of Radiology, Second Xiangya Hospital, Central South University, Changsha, 410011, China; 5 Department of Radiology, Wayne State University, Detroit, 48201, MI, United States of America; Henry Ford Health System, UNITED STATES

## Abstract

**Objectives:**

To evaluate the feasibility of differentiating between hepatocellular carcinomas (HCC) and healthy liver using diffusion tensor imaging (DTI).

**Material and Methods:**

All subjects underwent an abdominal examination on a 3.0T MRI scanner. Two radiologists independently scored the image quality (IQ). An optimal set of DTI parameters was obtained from a group of fifteen volunteers with multiple b-values (100, 300, 500, and 800 s/mm^2^) and various diffusion-encoding directions (NED = 6, 9, and 12)using two way ANOVA analysis. Eighteen Patients with HCC underwent DTI scans with the optimized parameters. Fractional anisotropy(FA) and average apparent diffusion coefficient (ADC) values were measured. The differences of FA and ADC values between liver healthy region and HCC lesion were compared through paired *t* tests.

**Results:**

There were no significant changes in liver IQ and FA/ADC values with increased NED(*P* >0.05), whereas the liver IQ and FA/ADC values decreased significantly with increased b-values(*P* <0.05). Good IQ, acceptable scan time and reasonable FA/ADC values were acquired using NED = 9 with b-value of (0,300) s/mm^2^. Using the optimized DTI sequence, ADC value of the tumor lesion was significantly lower than that of the healthy liver region (1.30 ± 0.34×10^−3^ vs 1.52 ± 0.27×10^−3^ mm^2^/s, *P* = 0.013), whereas the mean FA value of the tumor lesion (0.42 ± 0.11) was significantly higher than the normal liver region (0.32 ± 0.10) (*P* = 0.004).

**Conclusion:**

Either FA or ADC value from DTI can be used to differentiate HCC from healthy liver. HCC lead to higher FA value and lower ADC value on DTI than healthy liver.

## Introduction

Hepatocellular carcinoma (HCC) is the fifth most common cancer in the world and the third most frequent cause of death amongst oncological patients[1]. Alone, it accounts for 70%–85% of the worldwide total liver cancer burden[1]. Diffusion-weighted imaging (DWI) has been used to evaluate HCC[2], [3], as it can provide complimentary information over conventional magnetic resonance imaging (MRI). Conventional MRIs (T1 and T2 weighted signal intensities) and post gadolinium MR imaging are excellent for obtaining anatomical details but provide no functional information. DWI is one of the functional MR imaging methods, which offers noninvasive indirect assessment of microscopic diffusion of water molecules and microcirculation. In addition, apparent diffusion coefficient (ADC) values acquired from DWI can be quantitatively measured to reflect histopathological tissue characteristics[3], [4]. However, ADC calculated from DWI fails to define the characteristics of diffusion in anisotropic environments[5]. Tumors, in particular, are known to be heterogeneous[6], but diffusion in tumors is isotropic or anisotropic, which is currently controversial[3, 5, 7].

Diffusion tensor imaging (DTI) is a MRI technique that reveals microstructural characteristics of biological tissue, which can detect the degree of diffusion in multiple dimensions by using additional gradients[8, 9].Compared to three gradient-directions applied to DWI, at least six or more gradient directions for every section in DTI are needed to calculate the diffusion tensor[10, 11]. Through providing additional information on anisotropy diffusion and total diffusion orientations, DTI can achieve more precise ADC calculation[12].In addition, fractional anisotropy (FA) values obtained by DTI can be calculated in addition to ADC values, which are useful in evaluating the scalar properties of the diffusion of extracellular water molecules[13].

DTI has been used predominantly for brain imaging to characterize the healthy and diseased tissues in brain white matter[14–20]. A few studies have investigated the application of liver DTI on staging and diagnosis of liver fibrosis and inflammation, distinguishing cysts, hemangiomas, and metastases of the liver[5, 8, 12, 21–23]. However, different organs and tissues have different diffusion characteristics, Bachir et al[24]demonstrate that the liver, unlike the brain and kidney, has an isotropic diffusion pattern, probably due to its randomly organized structures.The cells in HCC were densely packed due to existence of more organelles, membranes, and fibers within the malignant cells[25]. But diffusion in HCC is isotropic or anisotropic, which is still unknown. In addition, a clinical evaluation exploring the feasibility of DTI for HCC diagnosis has not yet been reported.

As we know, b-value is the key factor in DW images, which will affect imaging quality and ADC values. Diffusion-encoding directions (NED) are the number of diffusion-encoding gradient directions, which is the other important factor in DTI acquisition. As NED increases, more DW images are used for the calculation of diffusion tensor, resulting in more accurate diffusion tensor estimation and a higher signal-to-noise ratio (SNR), but at the expense of a longer scan time [10]. However, there is still no clear conclusion about different schemes for selection of NED and for b-values in the evaluation of liver DTI indices. Therefore, an optimal set of DTI parameters for HCC scanning is critical in clinical practice. In this study, we have developed a strategy investigating the optimized DTI parameters for HCC scanning and intensely evaluated the feasibility of differentiating between HCC and healthy liver using FA/ADC values calculated from DTI.

## Materials and Methods

### 2.1 Patient population

The study was approved by the ethics committee of Affiliated Hospital of North Sichuan Medical College. Healthy volunteers were asked to voluntarily participate in this study, there was no specific protocol or methodology on the selection of the participants of this study as this was a convenience sample. However, a verbal informed consent regarding the goals of the study and the willingness to participate was taken by the HCC participants as the study qualified as involving only “minimal risks" to participants and the procedure was approved by the ethics committee of Affiliated Hospital of North Sichuan Medical College.15 healthy volunteers (6 females, age range 21–35 years, mean age27.5 ± 4.6 years) without any history of liver disease or significant alcohol consumption and 18 consecutive patients (7 females; age range 29–68years, mean age 42.1± 13.4 years) with histopathologic diagnosis of HCC through biopsy were included in this study.

### 2.2 MR imaging technique

All examinations were performed on a 3.0 T MR scanner with a 50 mT/m maximum gradient length and 200 T/m/s maximum slew rate (Discovery MR 750; GE Medical Systems, Milwaukee, Wis.) using a 32-channel body array coil with sixteen anterior and sixteen posterior elements. All examinations were implemented in the supine position.

After a gradient echo localizer, DTI was acquired on the 15 healthy volunteers in axial orientation with breath-hold fat saturated single spin echo planar imaging with the following parameters: TR 2500 ms; TE 70.1 ms; bandwidth 250 Hz; section thickness = 7 mm; intersection gap = 0 mm; matrix = 256×192; NEX = 1; and FOV = 28–34 cm, Auto shim: on. Slices number:18.

The DTI acquisitions were repeated twelve times with NED = 6, 9 and 12 and b = values of (0,100), (0,300), (0,500), and (0,800) s/mm^2^ respectively. Acquisition of more encoding directions leads to an extended scan time with the same scan range. Encoding directions (6, 9 and 12) correspond to total acquisition time of 16s, 22s, 28s. A maximum of 12 diffusion encoding directions were chosen in this study to limit patient’s breath hold period to within a comfortable range (28s).

18 HCC patients underwent the following routine upper abdominal MR sequences and the optimized DTI sequence:

1. A FRFSE T2-weighted sequence in axial orientation. The parameters were: TE = 90–100 ms, the TR was determined by the frequency of respiration; matrix = 256×192 NEX = 3; FOV = 26–33 cm; section thickness = 5 mm; intersection gap = 0.5 mm.
2. A 3D LAVA-Flex MR performed during one breath-hold with the following parameters: TR/TE = 4.2 ms/2.6 ms, 1.3 ms; matrix = 320×384×224; FOV = 26–33 cm; section thickness = 5 mm; intersection gap = 0mm; fat suppression technique: two-point Dixon method[26]and bandwidth = 166.7 kHz while NEX = 0.69. The acquisition time for LAVA-Flex was 17s.
3. A dynamic enhanced image performed with axial 3D LAVA–Flex sequencing. Gadolinium chelate (Magnevist, Schering Guangzhou Co, China) was administered intravenously (0.2 mmol/L per kilogram of body weight) at approximately 3.5 mL/s using a double tube high-pressure injector (Spectris MR Injection System, Medrad Inc, USA) and was followed by a 20 mL saline solution flushed at the same speed. From the beginning of the injection, two arterial phase images were created in 19 seconds; two portal vein phase images were created in 60 seconds and one equilibrium phase image was obtained within 180 seconds[27].

The DTI data were acquired after those conventional T2WI (a), T1WI (b) sequences and before dynamic enhanced image (c).

### 2.3 MR image analysis

#### 2.3.1 IQ Analysis—Qualitative Analysis

Image quality of liver DTIs was evaluated independently by two radiologists (with 6 and 7 years of experience in interpreting abdominal MR images respectively).The two radiologists were blinded to the b-value of the DTIs. Overall DTI image quality for parenchyma structure discrimination, distortion, susceptibility artifacts and motion artifacts was assessed[11]. Visual assessment was performed in consensus reading using a five point scale on which 1 point indicated unacceptable quality, 2 points indicated poor quality, 3 points indicated fair quality, 4 points indicated good quality, and 5 points indicated excellent quality[28]. The inter-observer agreement at the presence of scores was obtained for each image, and consensus was reached on cases that were graded differently between and used in the final analysis. The effect of NED and b value on qualitative scores of liver DTI was analyzed using Two Way ANOVA as well as the statistical interaction of b-values and NEDs.

#### 2.3.1 IQ Analysis—Quantitative Analysis

**a. Definitions of DTI Indices:** An average diffusion coefficient along each direction was derived from the DW images, as follows[29]:

$$S\left(i\right)=S_{0}\times e^{-b_{i}\times ADC}$$

where *S*(i) is the signal intensity (SI) measured on the ithb-value image and *b*_i_ is the corresponding b-value. S_0_ is the exact SI for a b-value = 0s/mm^2.^

From all diffusion-weighted images, the general diffusion tensor was first diagonalized, and the yielded scalar invariants of the tensor, including diffusion eigenvalues *λ*_1_, *λ*_2_, *λ*_3_.The primary eigenvalue *λ*_1_ (axial or longitudinal diffusivity) was the largest restricted diffusivity and the secondary and tertiary eigenvalues *(λ*_2_, *λ*_3_) (and their average) reflect lower restricted diffusion orthogonal to V1 (radial or transverse diffusion)[30].The index related to diffusional anisotropy was FA,

$$FA=\frac{\sqrt{3}}{\sqrt{2}}\frac{\sqrt{{\left(\lambda_{1}-\bar{\lambda }\right)}^{2}+{\left(\lambda_{2}-\bar{\lambda }\right)}^{2}+{\left(\lambda_{3}-\bar{\lambda }\right)}^{2}}}{\sqrt{\lambda_{1}^{2}+\lambda_{2}^{2}+\lambda_{3}^{2}}}$$

with $\bar{\lambda }$ being defined as[31]

$$\bar{\lambda }=\frac{\lambda_{1}+\lambda_{2}+\lambda_{3}}{3}$$

The range of FA value is from 0 to 1. FA value = 1 means those structures allowing diffusion restricted only along a single direction, whereas structures allowing completely free or isotropically diffusion should result in a FA of 0.

**Fig 1:**
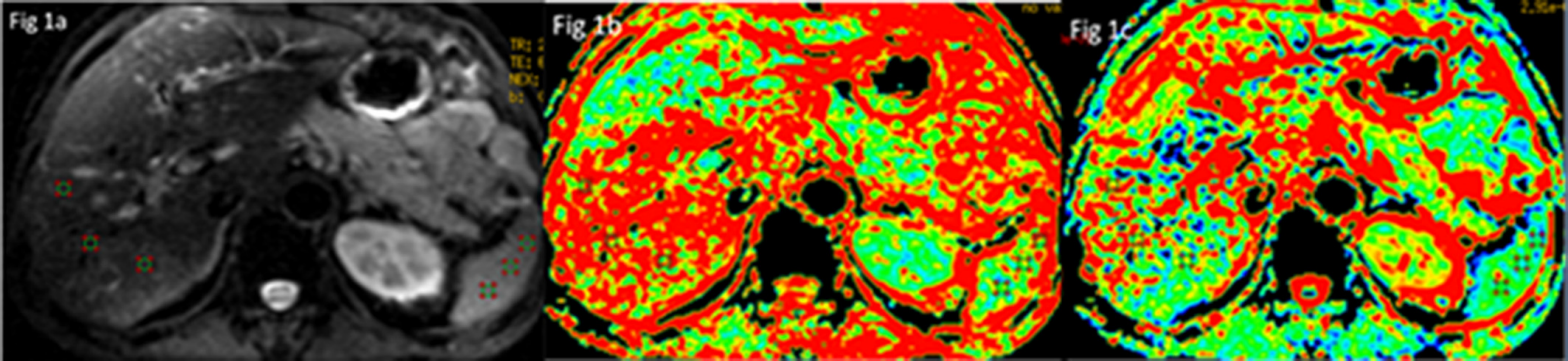
Three same ROIs placement of liver and spleen in signal intensity image (a), FA map (b) and ADC map(c).

**b. Measurements in normal subjects:** All the original MRI data were loaded onto a workstation (Advantage Workstation 4.4; GE Healthcare).The signal-to-noise ratio (SNR) of liver and contrast-to-noise ratio between liver and spleen (CNR(ls)) were calculated to quantitatively analyze IQ. By placing three circular 20 mm^2^ region-of-interests (ROIs) over the right lobe of the liver and spleen porta slice on signal intensity images with b = 0 with the aid of the T2-weighted images, then the three ROIs were mapped to the FA and ADC map (Fig 1).The mean SI value and standard deviation (SD) value were acquired for each tissue on signal intensity image. Vessels and bile ducts were carefully avoided when measuring. The SNR was defined as the SI divided by the standard deviation of each tissue including liver and spleen[32].The CNR(ls) was calculated using the following formula: CNR = SNR(liver)-SNR(spleen). Mean FA and ADC values were calculated from the ROIs on FA and ADC maps. The effect of NED and b value on SNR, CNR and FA/ ADC values was respectively analyzed using Two Way ANOVA as well as the statistical interaction of b-values and NEDs.

### 2.4 Optimization of liver DTI scan parameters

Two-Way ANOVA was used to analysis the effects of b-value and NED on IQ, FA and ADC values, as well as the statistical interaction of b-values and NEDs on each other. A set of optimized DTI scan parameters was determined based on good IQ, reasonable FA / ADC values and acceptable scan time. Because the acquisition of more encoding directions leads to an extended scan time, it would require patients to hold their breath for up to 28 seconds when choosing NED = 12 in this study; a task which many find extremely hard to complete.

### 2.5 Differentiation between HCC and healthy liver tissue using FA / ADC values

**Fig 2:**
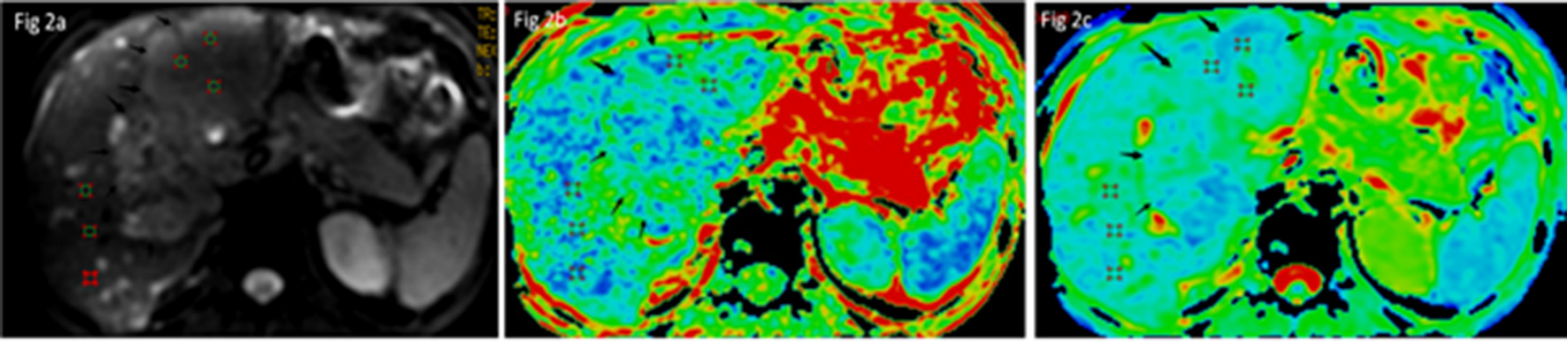
Three same ROIs placement over the most homogeneous part of each lesion and on the right liver lobe on the signal intensity image (a), FA map (b) and ADC map(c).

18 HCC patients underwent DTI scans using the optimized parameters. Both FA maps and ADC maps were created from the post-processing workstation. By placing three circular 50 mm^2^ ROIs over the most homogeneous part of each tumor lesion and the normal right liver lobe on the FA and ADC maps, the FA and ADC values of both HCC and healthy liver tissue were calculated (Fig 2). To ensure that the same areas were measured on the FA and ADC maps, the ROIs were first delineated on the DTI images with b = 0, and then mapped to the ADC and FA maps. To compare the differences of FA and ADC values of the liver lesions and normal right liver lobe, a paired t-test was used. All statistical analyses were performed using Minitab17.1.0 (State College, Pennsylvania). P values≤0.05 were considered indicative of a statistically significant difference.

## Results

Each volunteer was scanned successfully and no morphologic abnormalities were found on the images.

### 3.1 IQ—Qualitative assessment

**Fig 3:**
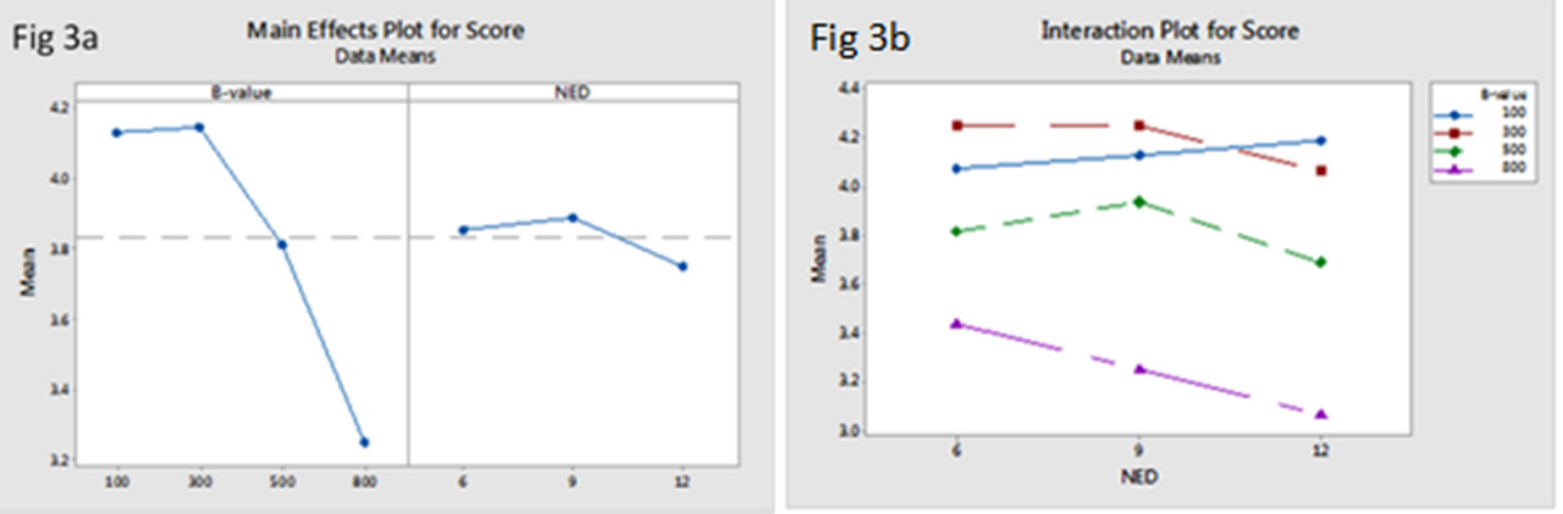
The main effect of b-values and NED on qualitative scoresof liver DTI (a) showed that the main effect of NED on qualitative scores of liver DTI was not significant, but the scores of the image quality of liver reduced with increased b-values. The interaction between b-values and NED on qualitative scores of liver DTI (b) showed that choosing b-value = 100, 300 s/mm^2^ with NED = 9 could gain the higher score.

Table 1 presents the results of the direct comparisons with different b-values and NED effects in terms of five-point-scale qualitative scores of liver DTI. The main effect of NED (6, 9, 12) on qualitative scores of liver DTI was not significant (F(2,83) = 1.23, *P* = 0.29), but the main effect of b-values (100, 300, 500, 800 s/mm^2^) was significant such that the scores of the image quality of liver reduced with increased b-values (F(3,83) = 25.36, *P* = 0.00) (Fig 3A).From analyzing the interaction between b-values and NED on qualitative scores of liver DTI (Fig 3B), choosing b-value of 100 and 300s/mm^2^ with NED = 9 as the set of new parameters could gain a similar higher score.

**Table 1:** Comparison of the Five-Point-Scale Qualitative Scores of liver DTI with different B values and NED Effects.

B-value NED	100 (s/mm2)	300 (s/mm2)	500 (s/mm2)	800 (s/mm2)
**6**	4.07(0.19)	4.15(0.48)	3.79(0.27)	3.50(0.41)
**9**	4.13(0.44)	4.12(0.23)	3.94(0.32)	4.25(0.46)
**12**	4.19(0.26)	4.19(0.45)	3.75(0.27)	3.00(0.80)

Note: The data are the mean image quality (standard deviation), Significant differences (*P*<0.05) are indicated with *. The main effect of NED (6, 9, 12) on qualitative scores of liver DTI was not significant (F (2, 83) = 1.23, *P* = 0.29), but the main effect of b-values (100, 300, 500, 800 s/mm^2^) was significant (F (3, 83) = 25.36, *P* = 0.00).

### 3.2 IQ—Quantitative assessments

#### 3.2.1 SNR and CNR(sl)

**Fig 4:**
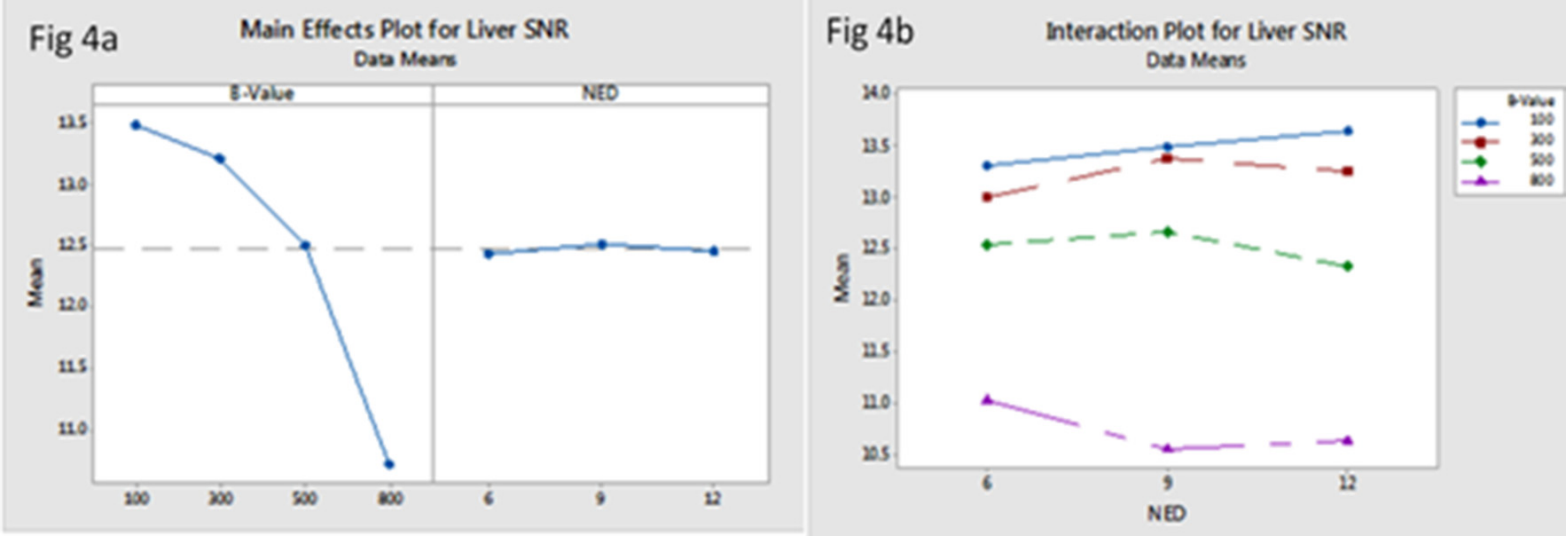
The main effect of b-values and NED on liver SNR (a) showed that the liver SNR reduced with increased b-values, but the differences had no significant with increased NED. The interaction between b-values and NED on liver SNR(b) showed that selected NED = 9 and b‑values range between 100 and 300s/mm^2^ lead to higher SNR of the liver DTI.

Table 2 shows the results of the direct comparisons with different b-values and NED in right liver SNR. The SNR significantly reduced with increased b-values (*F* (3, 80) = 3.21, *P* = 0.03), but there was no statistically significant difference in SNR with different NED (6, 9, 12) (*F* (2, 80) = 0.73, *P* = 0.48) (Fig 4A). By studying the interaction between b-values and NED on liver SNR (Fig 4B), we found that the highest SNR was selecting NED = 12 and b-values of 100 s/mm^2^, but NED = 9 and b‑values of 300 s/mm^2^ also lead to high SNR in the liver region on the DTI.

**Table 2:** Effects of b‑values and NED on liver SNR.

B-value NED	100 (s/mm2)	300 (s/mm2)	500 (s/mm2)	800 (s/mm2)
**6**	13.30(3.37)	12.99(3.24)	12.54(1.82)	11.02(3.15)
**9**	13.48(2.75)	13.37(4.18)	12.66(3.43)	10.55(1.60)
**12**	13.65(3.54)	13.25(3.46)	12.32(2.76)	10.62(3.22)

Note: The data are the mean value (standard deviation). Significant differences (*P*<0.05) are indicated with *. The SNR significantly reduced with increased b-values (*F* (3, 80) = 3.21, *P* = 0.03), but there was no statistically significant difference in SNR with different NED (6, 9, 12) (*F* (2, 80) = 0.73, *P* = 0.48).

In the spleen region of the DTI, the main effect of NED on SNR was not significant as well(*F*(2, 83) = 0.93, *P* = 0.39), while the main effect of b-values on SNR was significant such that the SNR significantly reduced with increased b-values (*F* (3, 83) = 17.49, *P* = 0.00). Moreover, there were no statistically significant differences in CNR(sl) with different NED and b-values((*F* (3, 83) = 0.24, *P* = 0.86), (*F* (2, 83) = 1.98, *P* = 0.15), respectively).

#### 3.2.2 FA and ADC values

**Fig 5:**
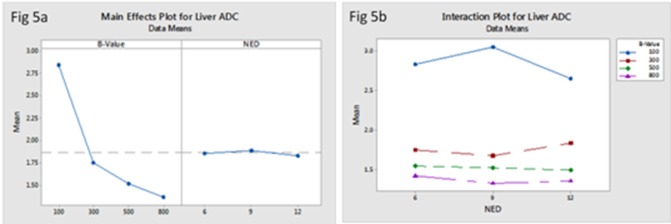
The main effect of b-values and NED on liver A DC (a) showed that the main effect of NED on liver ADC value was not significant, but liver ADC value reduced with increased b-values. The interaction between b-values and NED on liver ADC (b) showed that choosing b-value = 100s/mm^2^ no matter what values the NED were, the liver ADC values were the highest.

**Fig 6:**
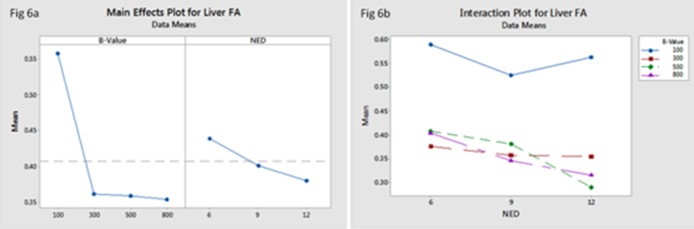
The main effect of b-values and NED on liver FA (a) showed that liver FA value reduced with increased b-values, even though the FA value of liver slightly reduced with increased NED, there was no statistically significant difference. The interaction between b-values and NED on liver FA (b) showed that selected b-value = 100s/mm^2^ no matter what values the NED were, theliver ADC values were the highest.

The FA and ADC values of liver significantly reduced with increased b-values (*F* (3, 83) = 22.86, *P* = 0.00; *F* (3, 83) = 90.17, *P* = 0.00respectively). However, there was no statistically significant difference in liver ADC values with different NED (Table 3), even though the FA values of liver slightly reduced with increased NED, there was no statistically significant difference (*F* (2, 83) = 0.29, *P* = 0.75) (Table 4) (Figs 5A and 6A). From the interaction map between b-values and NED on FA and ADC values of liver, we found that choosing b-value = 100s/mm^2^ no matter what values the NED were, both FA and ADC values of liver were the highest (Fig 5B) (Fig 6B).

**Table 3:** Effects of b‑values and NED on liver ADC.

B-value NED	100 (s/mm2)	300 (s/mm2)	500 (s/mm2)	800 (s/mm2)
**6**	2.83(0.27)	1.73(0.32)	1.51(0.11)	1.40(0.12)
**9**	3.05(0.80)	1.70(0.26)	1.54(0.20)	1.33(0.14)
**12**	2.65(0.52)	1.74(0.23)	1.46(0.17)	1.38(0.16)

Note: The data are the mean value (standard deviation). The ADC values were equal to mean value×10−3mm^2^/s. Significant differences (*P*<0.05) are indicated with *. The ADC values of liver significantly reduced with increased b-values *F* (3, 83) = 90.17, *P* = 0.00). However, there was no statistically significant difference in liver ADC values with different NED (F (2, 83) = 0.29, *P* = 0.75).

**Table 4:** Effects of b‑values and NED on liver FA.

B-value NED	100 (s/mm2)	300 (s/mm2)	500 (s/mm2)	800 (s/mm2)
**6**	0.59(0.14)	0.38(0.05)	0.43(0.09)	0.42(0.06)
**9**	0.53(0.11)	0.37(0.07)	0.38(0.09)	0.34(0.04)
**12**	0.56(0.19)	0.36(0.10)	0.29(0.06)	0.33(0.05)

Note: The data are the mean value (standard deviation). Significant differences (*P*<0.05) are indicated with *. The FA values of liver significantly reduced with increased b-values *F* (3, 83) = 20.36, *P* = 0.00). However, there was no statistically significant difference in liver ADC values with different NED (F (2, 83) = 3.14, *P* = 0.05).

### 3.3 The Optimized liver DTI scan parameters

**Fig 7:**
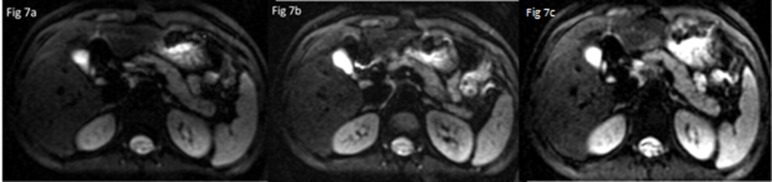
Comparison of image quality among of different NEDs: NED = 6 (a), NED = 9(b) and NED = 12(c) show equally good image quality (five points).

**Fig 8:**
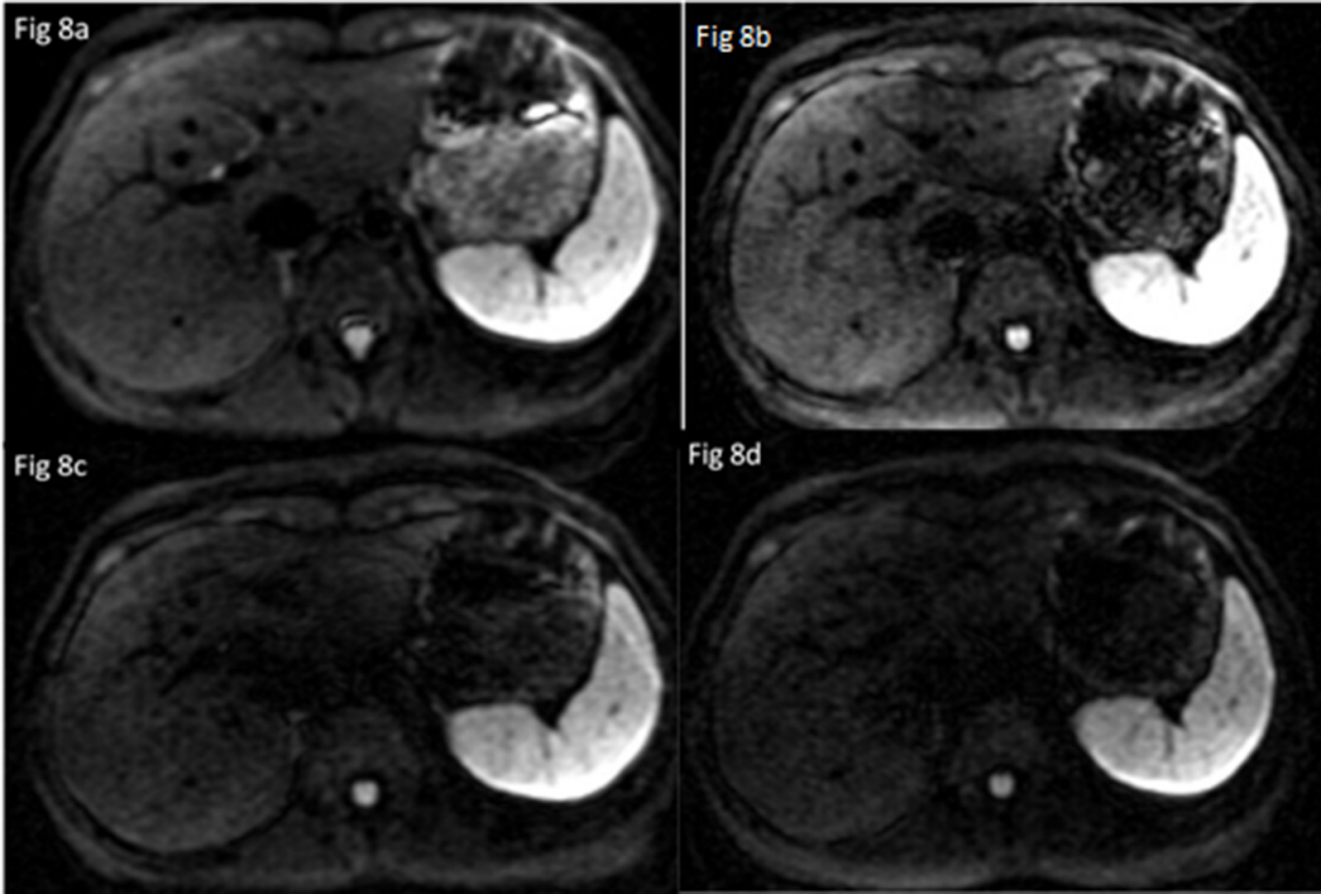
Comparison of image quality among different b-values 100s/mm^2^ (a), 300s/mm^2^ (b),500s/mm^2^ (c), 800s/mm^2^ (d). Both readers judged that the liver signal intensity was markedly reduced and the noise was significantly increased with increased B values, especially b-value = 800s/mm^2^, it was difficult to discriminate the boundry of vascular structures in left liver.

The optimized b-value and number of encoding directions were obtained by both qualitative and quantitative image analysis, which mainly depended on good imaging quality, acceptable scan time and reasonable FA/ADC values. In this study, we found that the main effect of b-values (100, 300, 500, 800 s/mm^2^) on among IQ, FA and ADC values of liver DTI was significant such that the qualitative scores, SNR, ADC and FA of liver reduced with increased b-values (Fig 7), however, the main effect of NED (6, 9,12) on IQ and ADC/FA values of liver DTI was not significant (Fig 8). Moreover, choosing b-value = 100 and 300 s/mm^2^ with NED = 9 as the set of new parameters could gain the higher qualitative score of the liver DTI. Even though selecting NED = 12 and b-values of 100 s/mm^2^ lead to the highest SNR, but it will result in 28second scan time and increase the effect of perfusion. In addition, we found that choosing b-value = 100s/mm^2^ no matter what values the NED were, both FA and ADC values of liver were the highest, we speculated that the reason was due to the perfusion effects in an overestimation of FA and ADC value. Moreover, when choosing NED = 9, the acquisition time was 22second.Therefore, with regard to the above results, the best compromise among acquisition time, resulting image quality and reasonable FA/ADC values seems the application of NED = 9 with b-values = 300 s/mm^2^ for liver DTI.

### 3.4 Differences between normal liver and liver lesions using FA/ADC values

**Fig 9:**
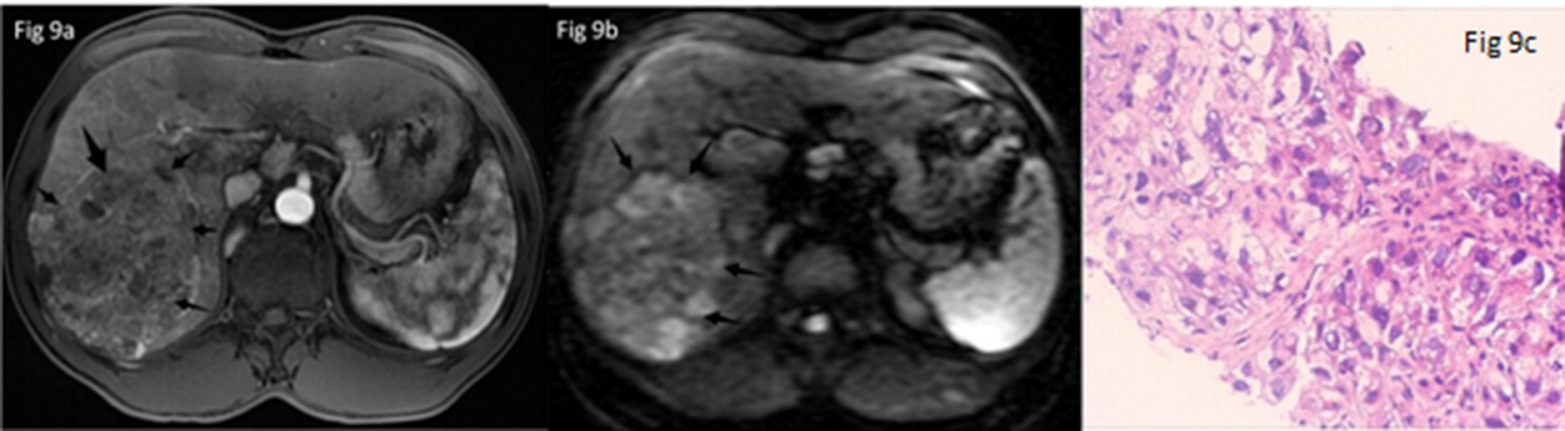
A patient with right liver lobe primary liver cancer. The tumor appeared hyperintense on DTI image (a). On dynamic gadolinium-enhanced imaging it showed markedly inhomogeneous enhancement at arterial phase (b). Surgical histopathologic diagnosis (c) confirmed it HCC. The tumor had an ADC value of 1.27×10^−3^mm^2^/s and a FA value of 0.33, respectively.

The 18 HCC patients all underwent the new DTI sequence (with NED = 9, b value = 300s/mm2).The mean FA value of HCC lesions (0.42 ± 0.11) was significantly higher than that of normal right liver (0.32 ± 0.10) (*P* = 0.004). The ADC values of HCC lesions and normal right liver were 1.30±0.34×10^−3^mm^2^/s vs 1.52± 0.27×10−3mm2/s, also demonstrating a significant difference (*P* = 0.013). (Table 5) (Fig 9).

**Table 5:** Comparison of the FA and ADC value between HCC lesions and normal liver.

Parameter	HCC lesions	Normal liver	P Value
ADC value	1.30 (0.34)	1.52 (0.27)	0.013*
FA value	0.42(0.11)	0.32 (0.10)	0.004*

Note: The data are the mean ADC and FA ((standard deviation). The ADC values were equal to mean value×10−3mm^2^/s. Significant differences (*P*<0.05) are indicated with *.

## Discussion

An optimal set of MRI acquisition parameters is critical for clinical diagnosis since it significantly affects the MRI quality and acquisition time. The major findings of this study are: 1) There were no significant changes in liver IQ and FA/ADC values with increased NED(*P* >0.05).2)The liver IQ and FA/ADC values decreased significantly with increased b-values(*P* <0.05). 3) Among acquisition time, resulting image quality and reasonable FA/ADC values seems to be the application of 9 encoding directions with b-values of 300 s/mm^2^ as a set of parameters for liver DTI on GE 3.0 T. 4)DTI with FA and ADC values can be used to different HCC from healthy liver.

The diffusion induced signal attenuation in DTI was measured for water displacement along at least six non collinear directions[11]. Xia et al and Cosottini et al claimed that more directions scanned led to better recording with longer scan time [23, 33].However, Hasan et al [9] declared that there was no advantage to using more than 6 sampling orientations as long as the selected orientations point to the vertices of an icosahedron. Chuck NC [11] similarly found that FA values in the cortex of kidney showed no significant difference between encoding directions of 6 and 32. Our results were consistent with Hasan and Chuck’s results although our choices of NEDs were different. Based on our study, NED (6, 9, 12) does not exhibit a significant effect on imaging quality and FA/ADC values of liver DTI (*P*>0.05). It is notable that we chose smaller NEDs than the above referenced works. Large NEDs (>12) are suitable for brain DTI scans since no breath holding technique is required. However, for moving organs like liver, a breath holding technique should be adopted, especially because a respiratory gating technique is not allowed to use in liver DTI on 3.0T GE and when patient breathing pattern is irregular. Moreover, irregularities in breathing may lead to increased motion artifacts and mis-registration in liver DTI. Therefore, only NED of 6 (16sec), 9 (22sec) and 12 (28sec) were considered in our study. Although the main effect of NED (6, 9, 12) on imaging quality and FA/ADC values of liver DTI was not significant, our statistical interaction analysis indicated a combination of NED = 9 (22sec) with b-value = 100, 300 s/mm^2^ gain a higher qualitative score and a higher SNR of the liver DTI which was the main reason for us to pick NED = 9 as the optimized DTI scan parameter. It is well recognized that sequences scanned with higher b-values are considered to be more diffusion-weighted than sequences with lower b-values, while at an expense of a lower SNR[34]. Our findings were in line with previous reports[34–36] that image quality (qualitative score& SNR), FA/ADC values of liver reduced with increased b-values (100, 300, 500, 800 s/mm^2^).However, overly low b-values might result in an overestimation of FA/ADC values due to perfusion effects when applying higher b-values; the faster spin ensembles are already dephased and cannot contribute to the signal[11]. B Chuck NC[11]proved in her kidney study that the optimal b‑value for DTI should range from 300 to 500 s/mm^2^.Considering both quantitative and qualitative aspects, b-value = 300s/mm^2^ was picked as the optimized value for our patient liver DTI scans to gain better image quality without overestimation of FA/ADC values.

DTI provides more accurate ADC calculation than DWI[12]. Low ADC implies that water mobility is restricted, indicating cells are densely packed and a high ADC implies that water can move freely, indicating low cellularity[37]. Based on our experiments on patient’s DTI data, HCC lesions presented significant lower ADC values than normal liver (P = 0.013) which is in line with previous ADC studies on DWI [24, 38].Moreover, DTI provides FA value which is used to characterize thedegree of diffusion anisotropy. Kinoshita et al[39] reported that FA exhibited a strong correlation with cell density (*R* = 0.75). Sugita et al[25] demonstrated the cells in malignant lesions were densely packed due to existence of more organelles, membranes, and fibers within the malignant cells. Both studies suggest higher FA indicated higher tumor cell density and higher malignant potential. Szczepankiewicz, F et al [40] claimed the FA value of meningioma and the glioblastoma was lower than normal brain tissues, but Erturk, S. M et al[5]found that metastases in liver have high FA values. We found that HCCs have higher FA values, which is not consistent with the former findings, we speculated that the reason was due to different tissues structures and different histological types of tumor cells.

Through thoroughly investigating the effect of NED, b value to image quality and FA/ADC value, we presented an optimal parameter setting for clinical DTI liver scan. In addition, FA/ADC values were found to be great indicators for HCC diagnosis which exhibits great value to clinical practice. Future work will focus on grading of HCC based on FA/ADC values. However, a small number of HCC patients in this study may introduce a slightly biased conclusion for further analysis. A large scale study is underway to evaluate the feasibility of grading HCC using FA/ADC values from liver DTIs.

## Conclusion

The best compromise among acquisition time, resulting image quality and reasonable FA/ADC values is NED = 9 with b-values of 300s/mm^2^ as the set of new parameters for liver DTI. FA and ADC from DTI could be used as indexes to differentiate HCC from healthy liver.

## Supporting Information

S1 Fig Example of measurements in normal subjects. Three same ROIs placement of liver and spleen in signal intensity image (a), FA map (b) and ADC map(c).
(PDF)

S2 Fig Example of measurements in HCC patients. Three same ROIs placement over the most homogeneous part of each lesion and on the right liver lobe on the signal intensity image (a), FA map (b) and ADC map(c).
(PDF)

S3 Fig Qualitative assessmentof five-point-scale qualitative scores of liver DTI. The main effect of b-values and NED on qualitative scores of liver DTI (a) showed that the main effect of NED on qualitative scores of liver DTI was not significant, but the scores of the image quality of liver reduced with increased b-values. The interaction between b-values and NED on qualitative scores of liver DTI (b) showed that choosing b-value = 100, 300 s/mm^2^ with NED = 9 could gain the higher score.
(PDF)

S4 Fig Results of the direct comparisons with different b-values and NED in liver SNR. The main effect of b-values and NED on liver SNR (a) showed that the liver SNR reduced with increased b-values, but the differences had no significant with increased NED. The interaction between b-values and NED on liver SNR(b) showed that selected NED = 9 and b‑values range between 100 and 300s/mm^2^ lead to higher SNR of the liver DTI.
(PDF)

S5 Fig Results of the direct comparisons with different b-values and NED in liver ADC. The main effect of b-values and NED on liver A DC (a) showed that the main effect of NED on liver ADC value was not significant, but liver ADC value reduced with increased b-values. The interaction between b-values and NED on liver ADC (b) showed that choosing b-value = 100s/mm^2^ no matter what values the NED were, the liver ADC values were the highest.
(PDF)

S6 Fig Results of the direct comparisons with different b-values and NED in liver FA. (PDF)

S7 Fig Comparison of image quality among of different NEDs. NED = 6 (a), NED = 9(b) and NED = 12(c) show equally good image quality (five points).
(PDF)

S8 Fig Comparison of image quality among different b-values 100s/mm^2^ (a), 300s/mm^2^ (b),500s/mm^2^ (c), 800s/mm^2^ (d). Both readers judged that the liver signal intensity was markedly reduced and the noise was significantly increased with increased B values, especially b-value = 800s/mm^2^, it was difficult to discriminate the boundry of vascular structures in left liver.
(PDF)

S9 Fig A patient with right liver lobe primary liver cancer. The tumor appeared hyperintense on DTI image (a). On dynamic gadolinium-enhanced imaging it showed markedly inhomogeneous enhancement at arterial phase (b). Surgical histopathologic diagnosis (c) confirmed it HCC. The tumor had an ADC value of 1.27×10^−3^mm^2^/s and a FA value of 0.33, respectively.
(PDF)

S10 Fig Supporting Information Stard Checklist. (PDF)

S1 Table Comparison of the Five-Point-Scale Qualitative Scores of liver DTI with different B values and NED Effects. (PDF)

S2 Table Effects of b‑values and NED on liver SNR. (PDF)

S3 Table Effects of b‑values and NED on liver ADC. (PDF)

S4 Table Effects of b‑values and NED on liver FA. (PDF)

S5 Table Comparison of the FA and ADC value between HCC lesions and normal liver. (PDF)
