# Supplementary figures and images for: Preliminary Study of MR Diffusion Tensor Imaging of the Liver for the Diagnosis of Hepatocellular Carcinoma
Source: PLoS One. 2015 Aug 28;10(8):e0135568. doi: 10.1371/journal.pone.0135568 (PMC4552840; doi:10.1371/journal.pone.0135568)

**Fig1. Example of measurements in normal subjects.**

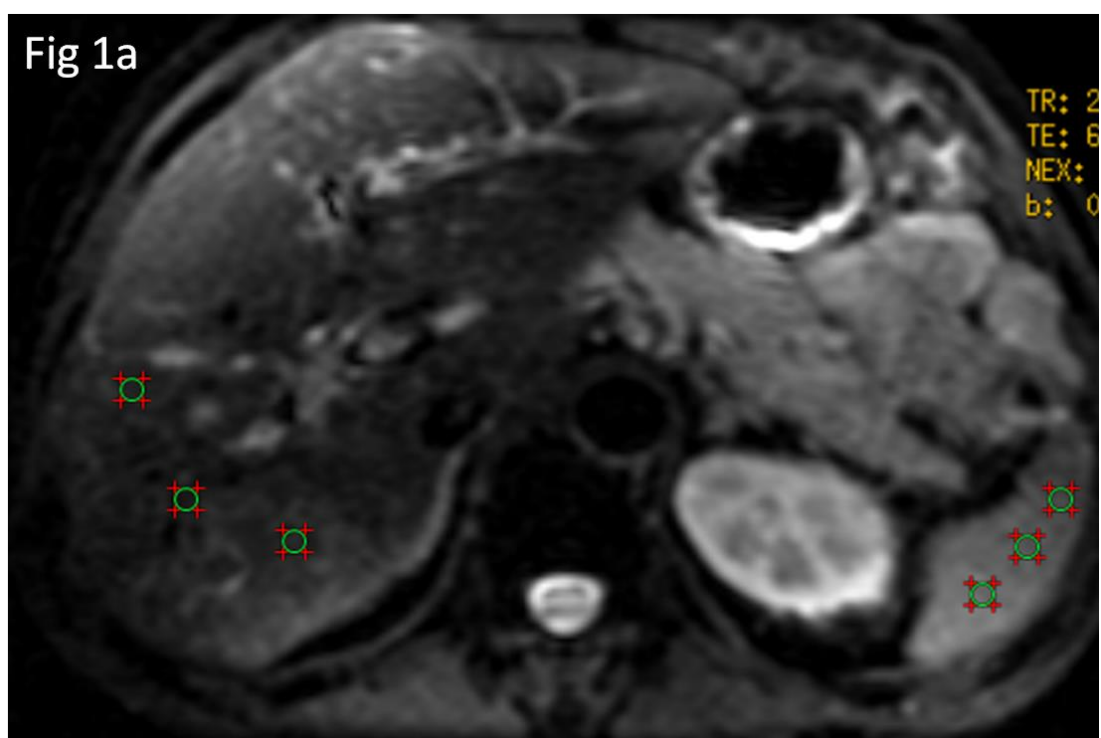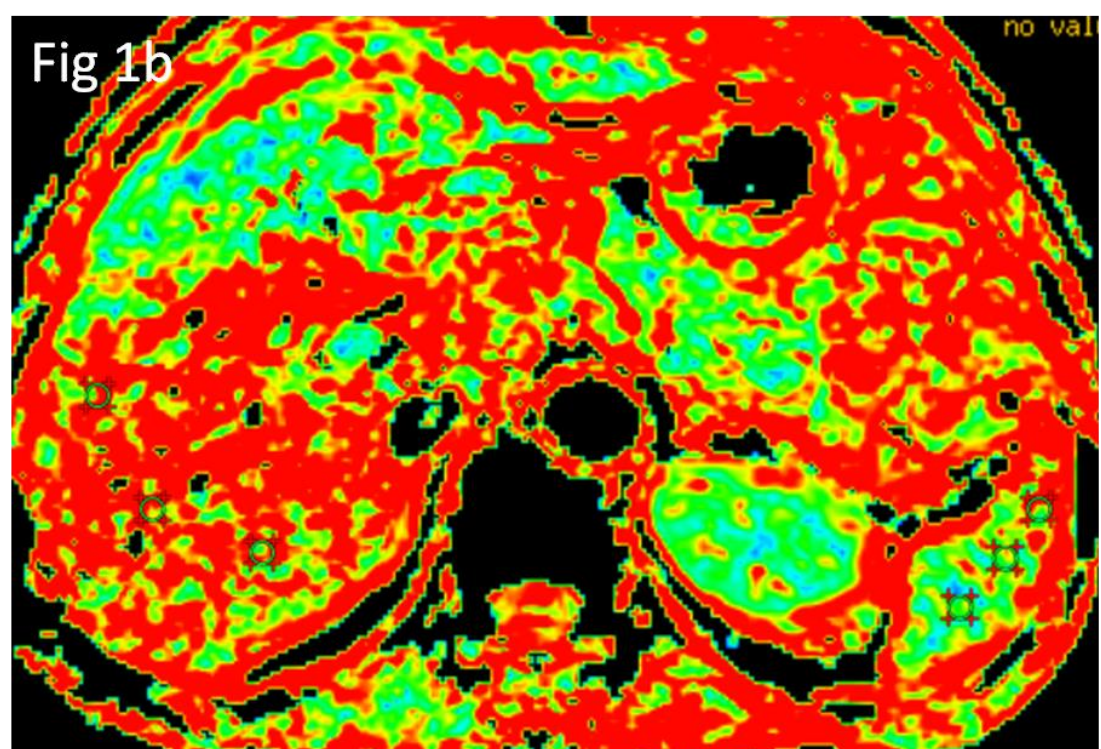

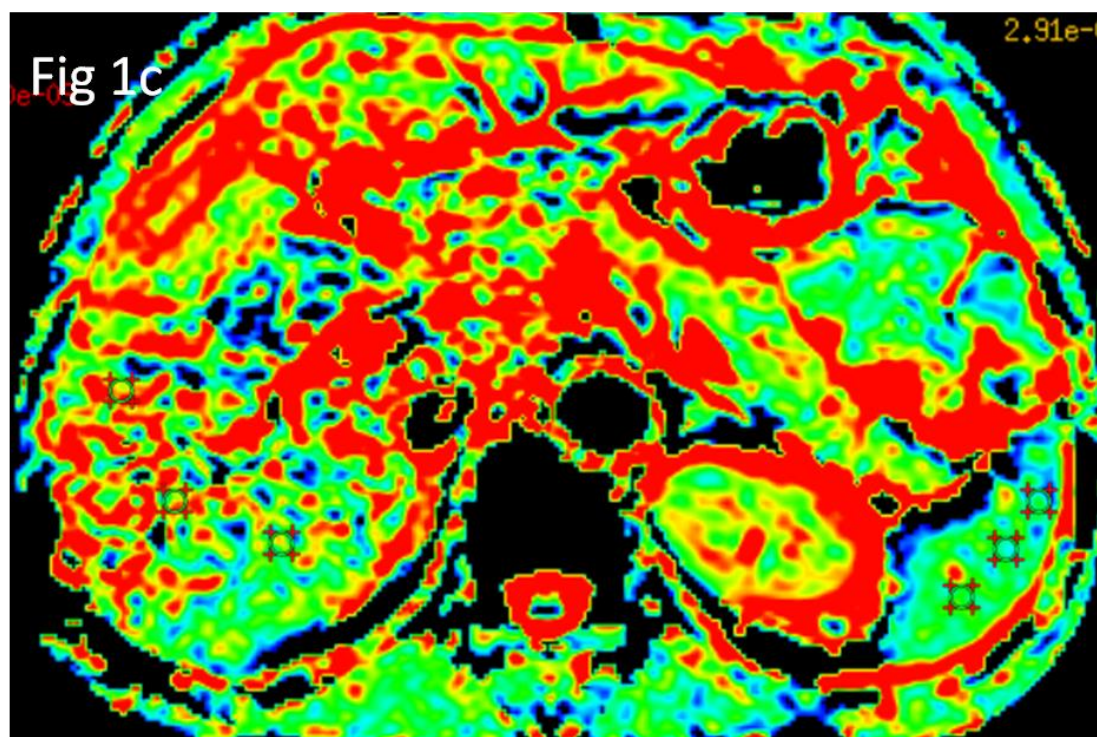

Supplement: S1 Fig — Three same ROIs placement of liver and spleen in signal intensity image (a), FA map (b) and ADC map(c). (PDF) [file pone.0135568.s001.pdf]

**Fig 2. Example of measurements in HCC patients.**

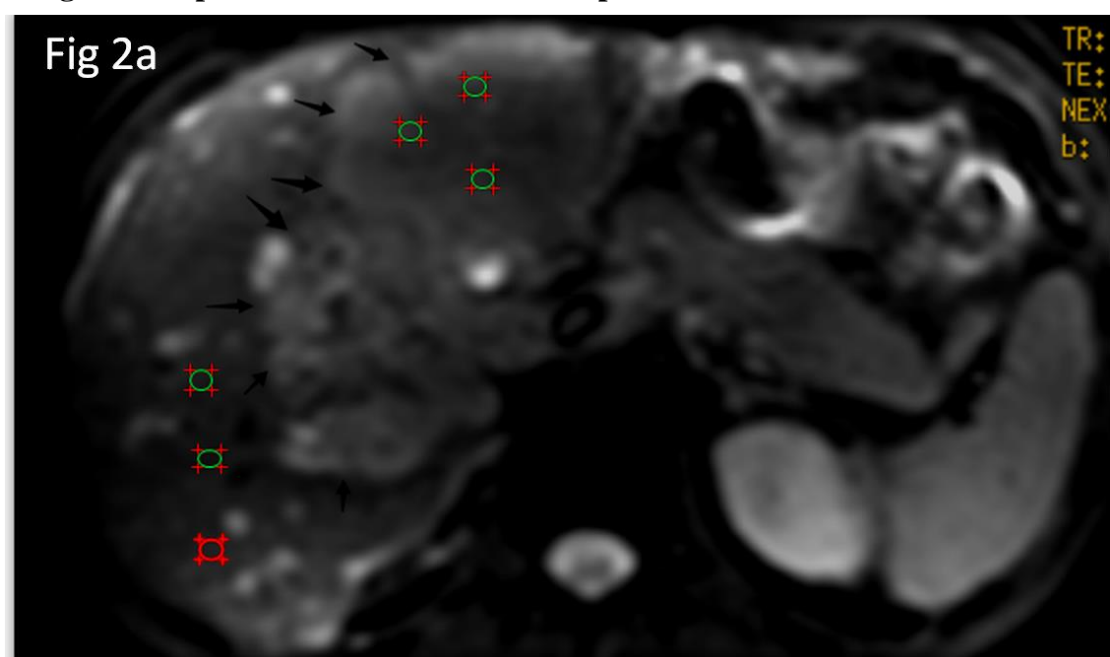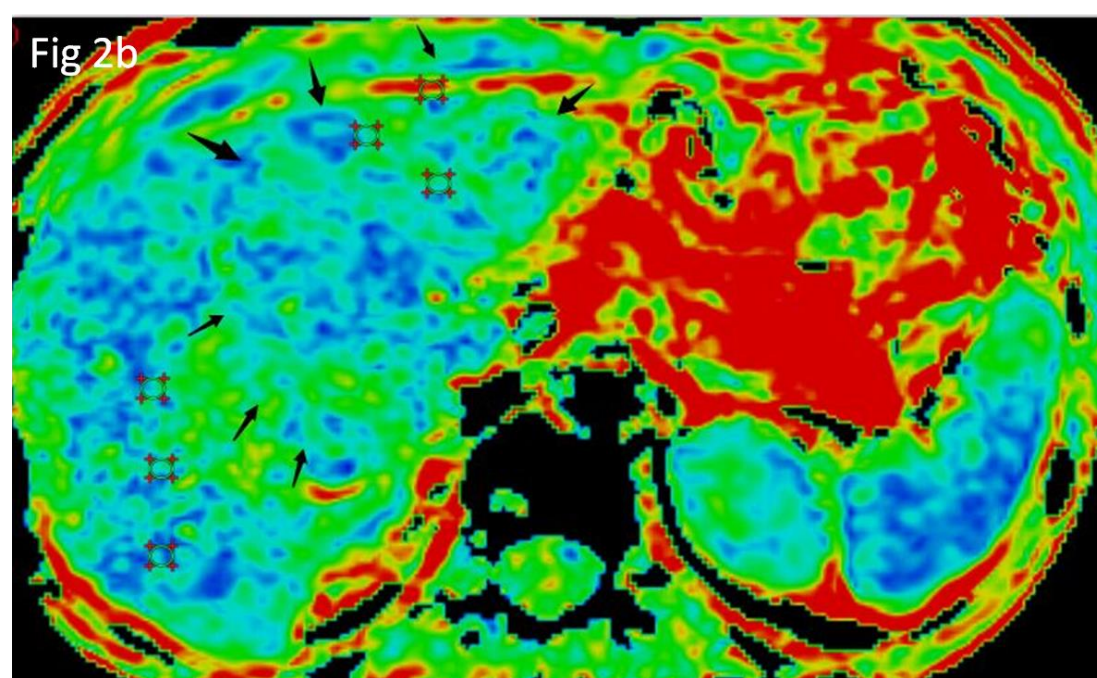

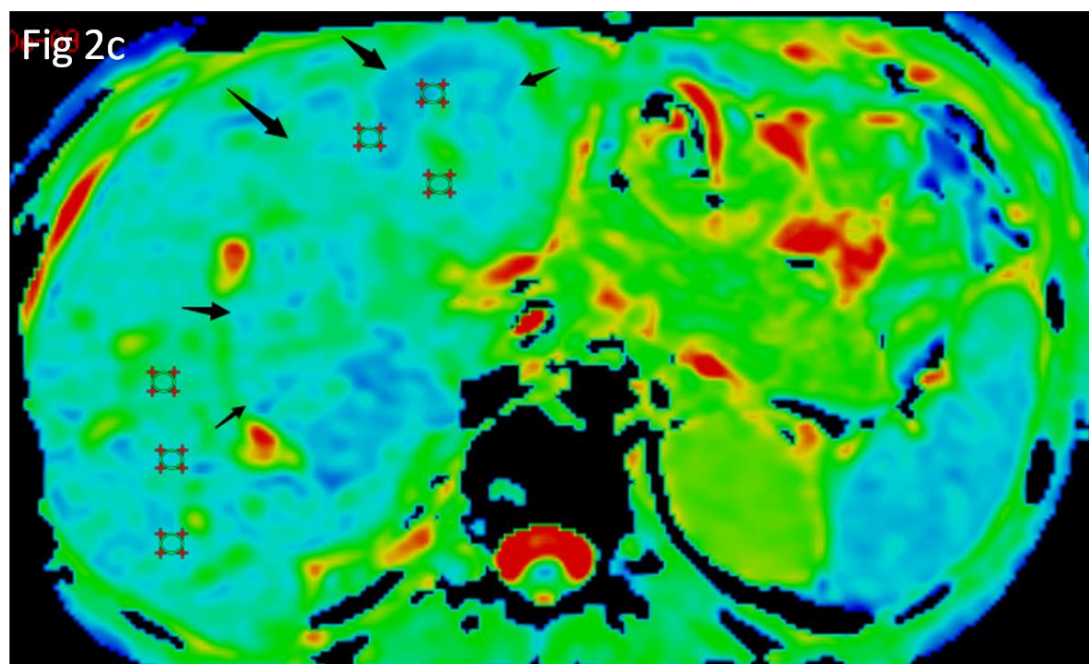

Supplement: S2 Fig — Three same ROIs placement over the most homogeneous part of each lesion and on the right liver lobe on the signal intensity image (a), FA map (b) and ADC map(c). (PDF) [file pone.0135568.s002.pdf]

**Fig 5. Results of the direct comparisons with different b-values and NED in liver ADC.**

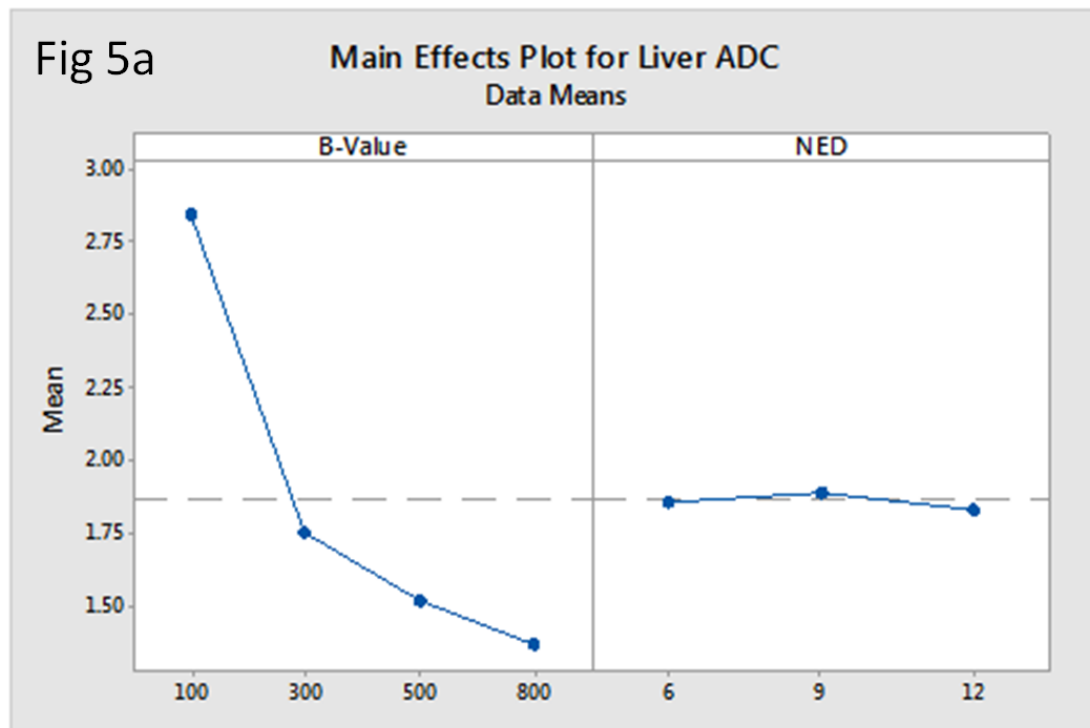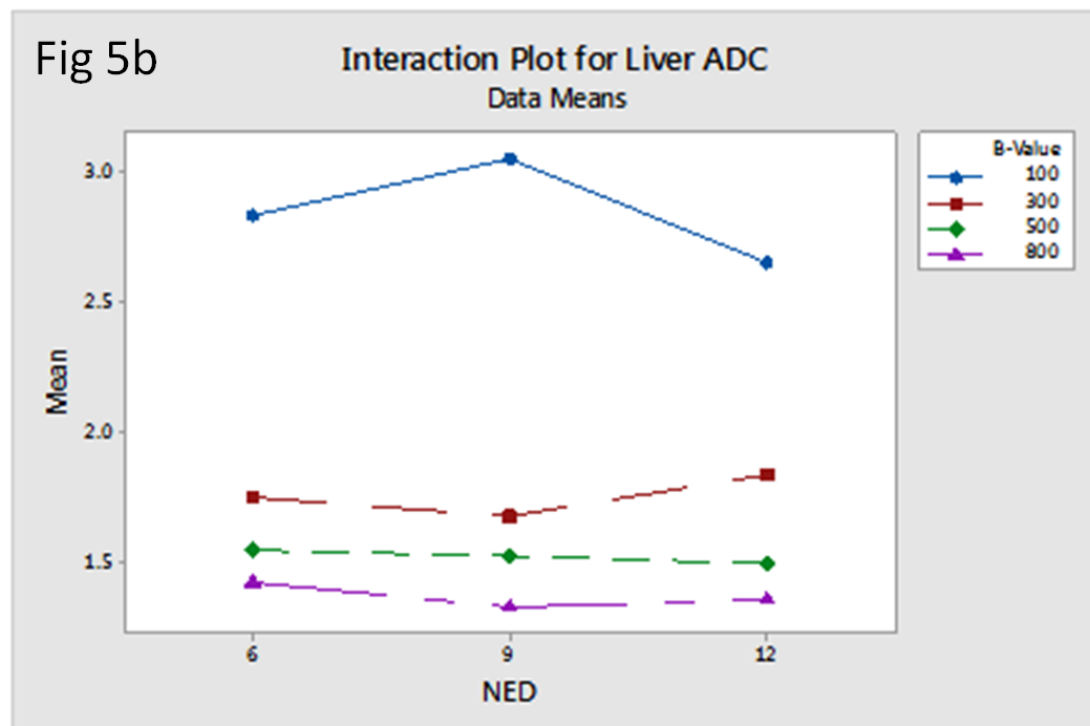

Supplement: S5 Fig — The main effect of b-values and NED on liver A DC (a) showed that the main effect of NED on liver ADC value was not significant, but liver ADC value reduced with increased b-values. The interaction between b-values and NED on liver ADC (b) showed that choosing b-value = 100s/mm2 no matter what values the NED were, the liver ADC values were the highest. (PDF) [file pone.0135568.s005.pdf]

**Fig 6. Results of the direct comparisons with different b-values and NED in liver FA.**

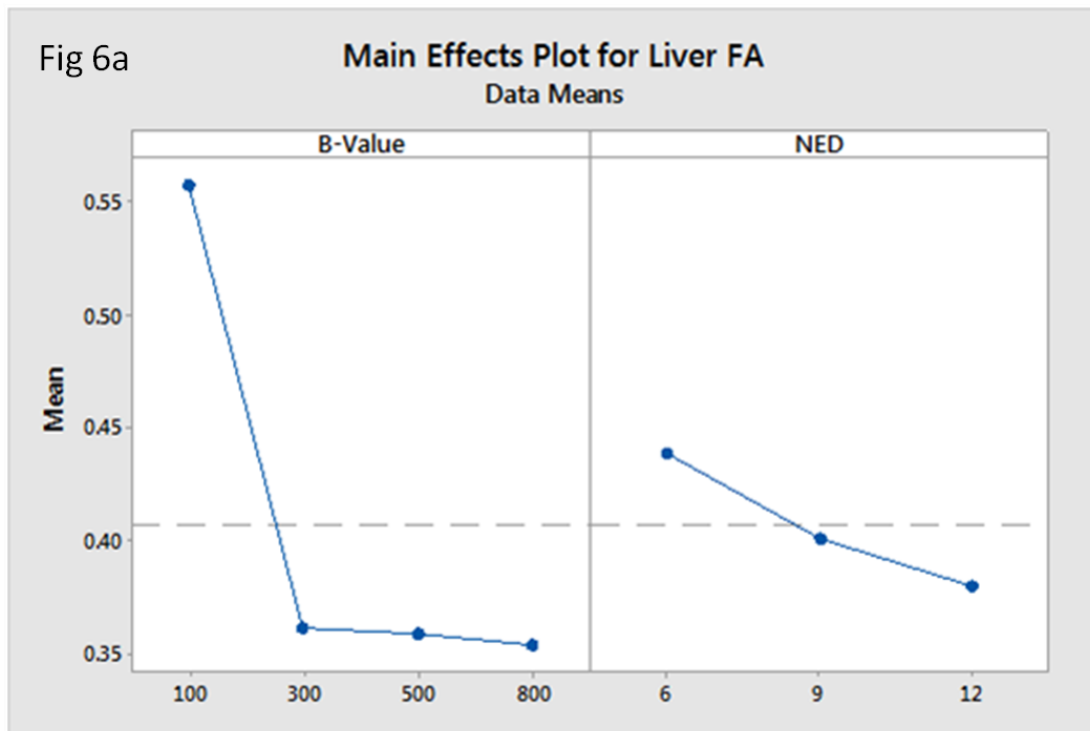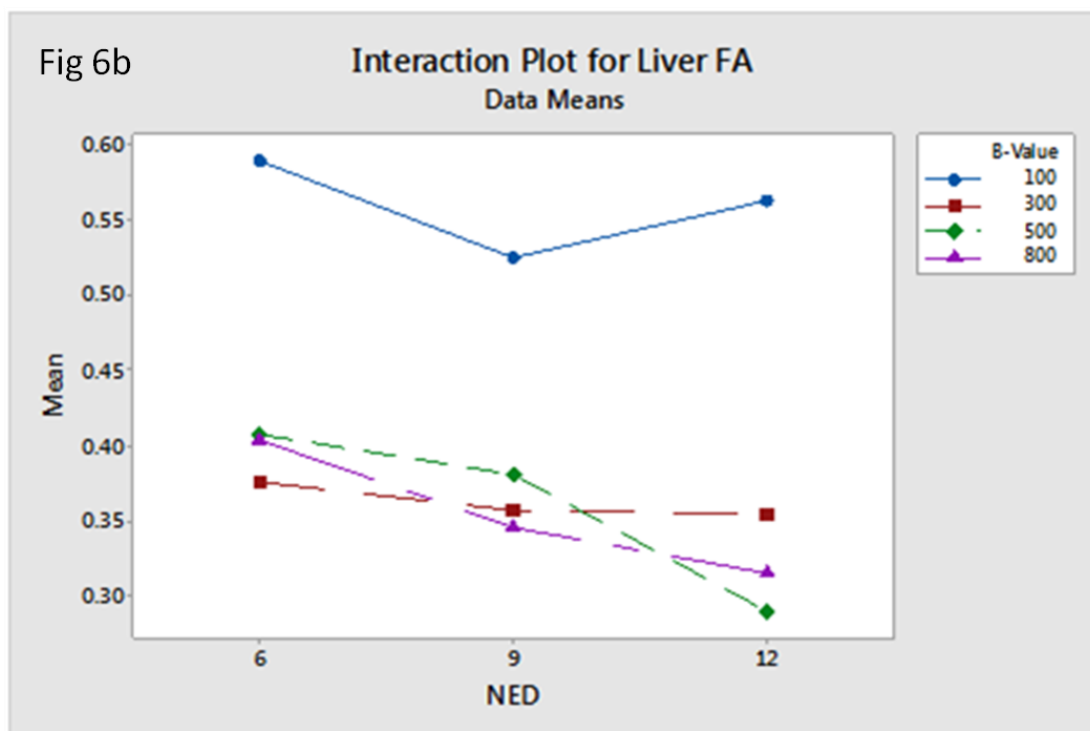

Supplement: S6 Fig — (PDF) [file pone.0135568.s006.pdf]

**Fig 7. Comparison of image quality among of different NEDs.**

**Fig 7a**

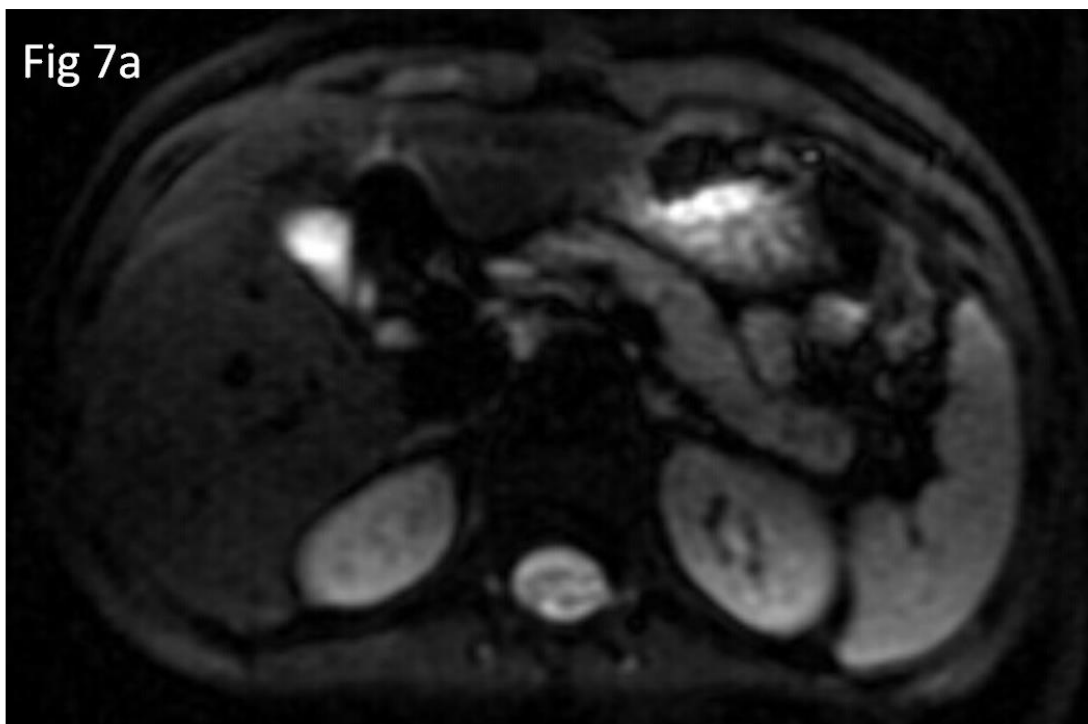

**Fig 7b**

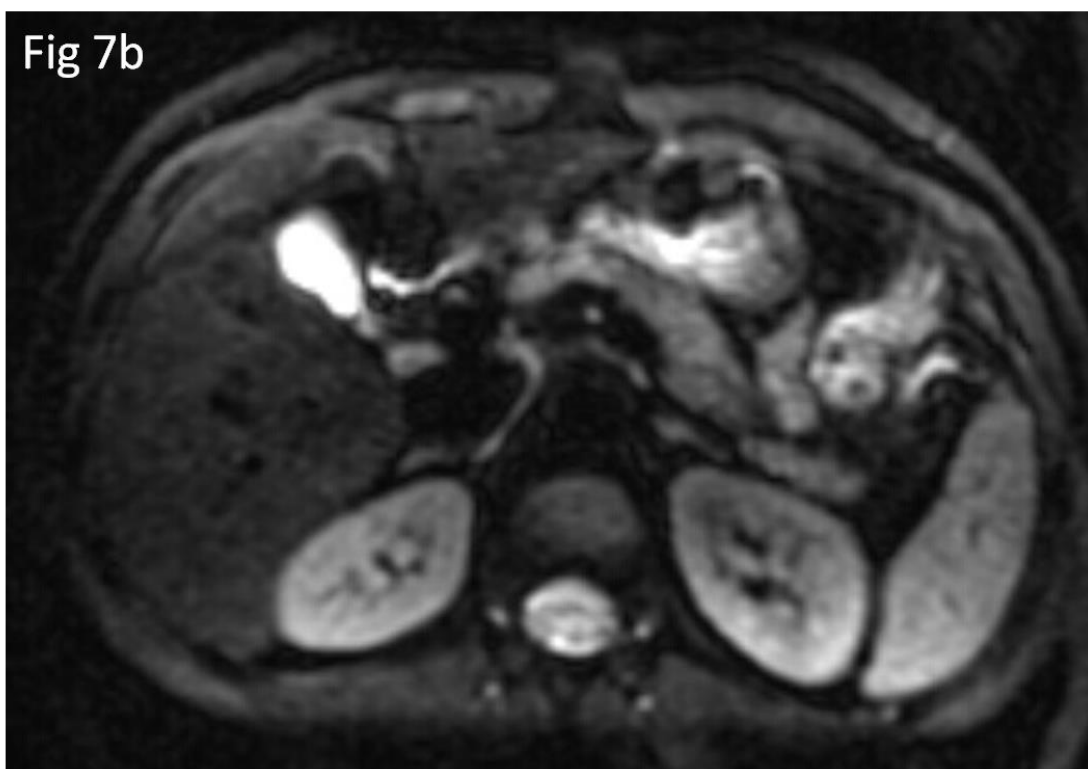

Fig 7c

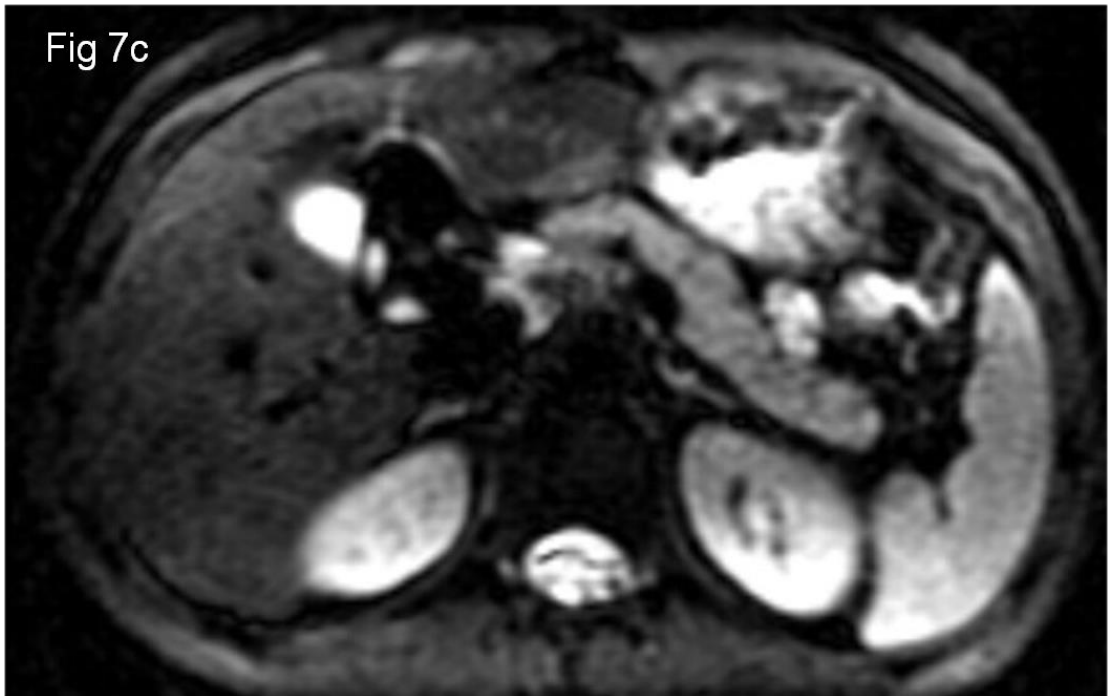

Supplement: S7 Fig — NED = 6 (a), NED = 9(b) and NED = 12(c) show equally good image quality (five points). (PDF) [file pone.0135568.s007.pdf]

**Fig 8. Comparison of image quality among different b-values.**

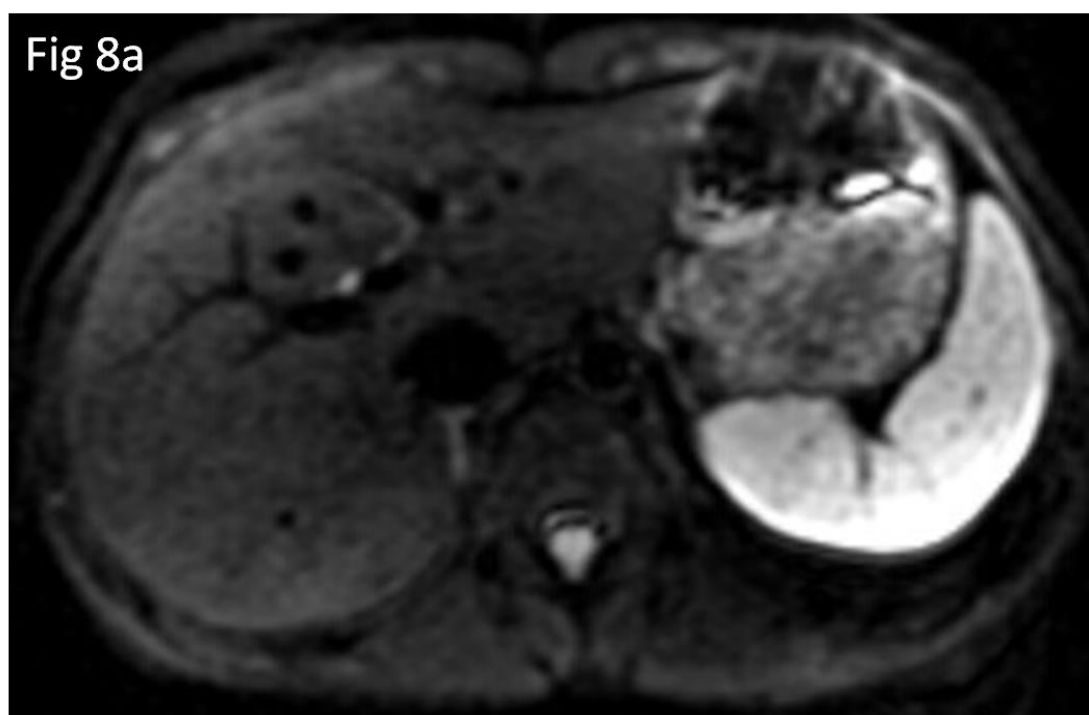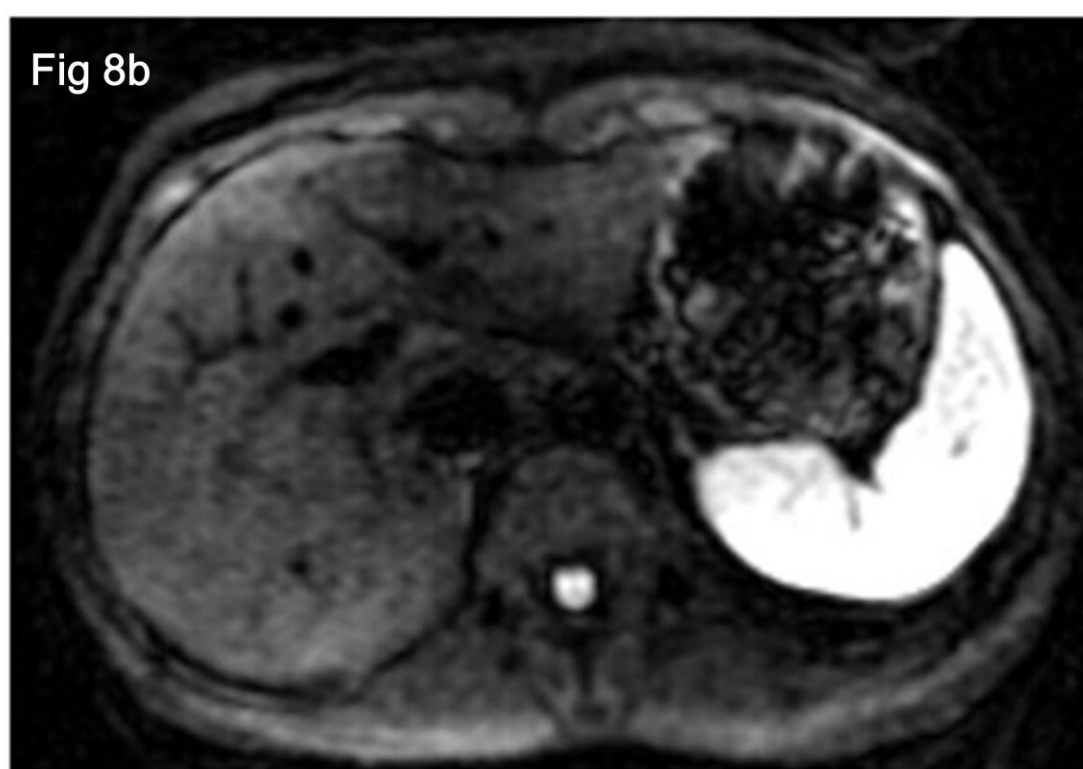

Fig 8c

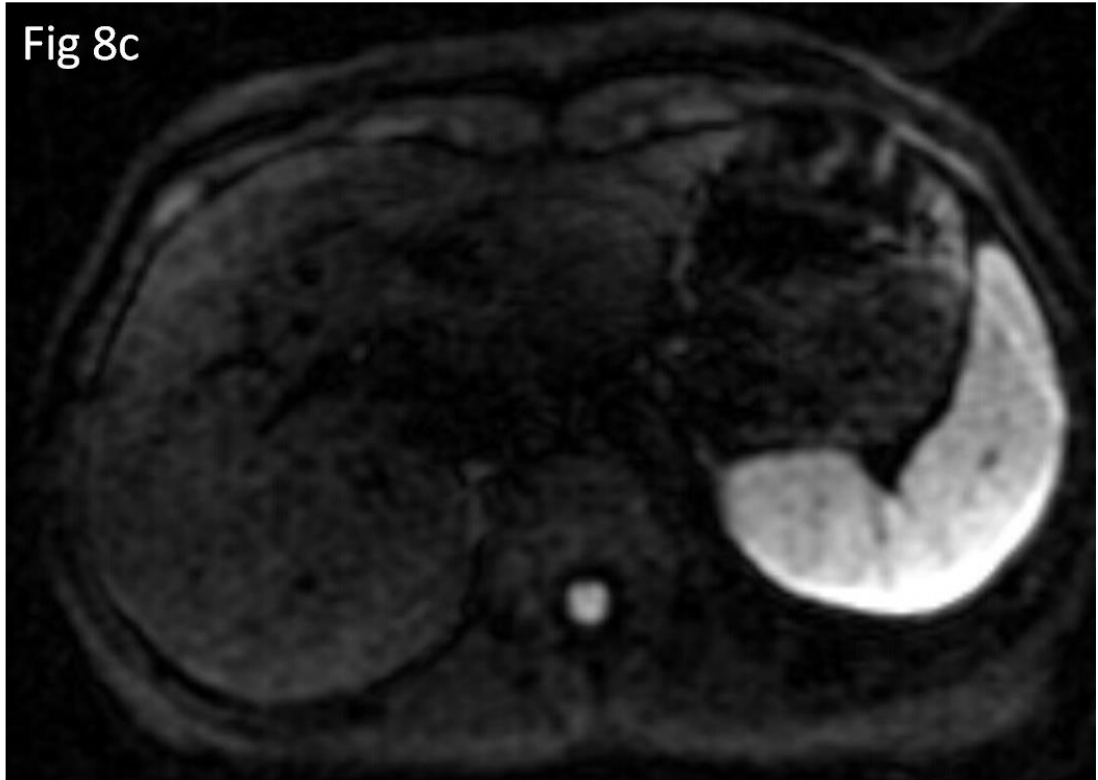

Fig 8d

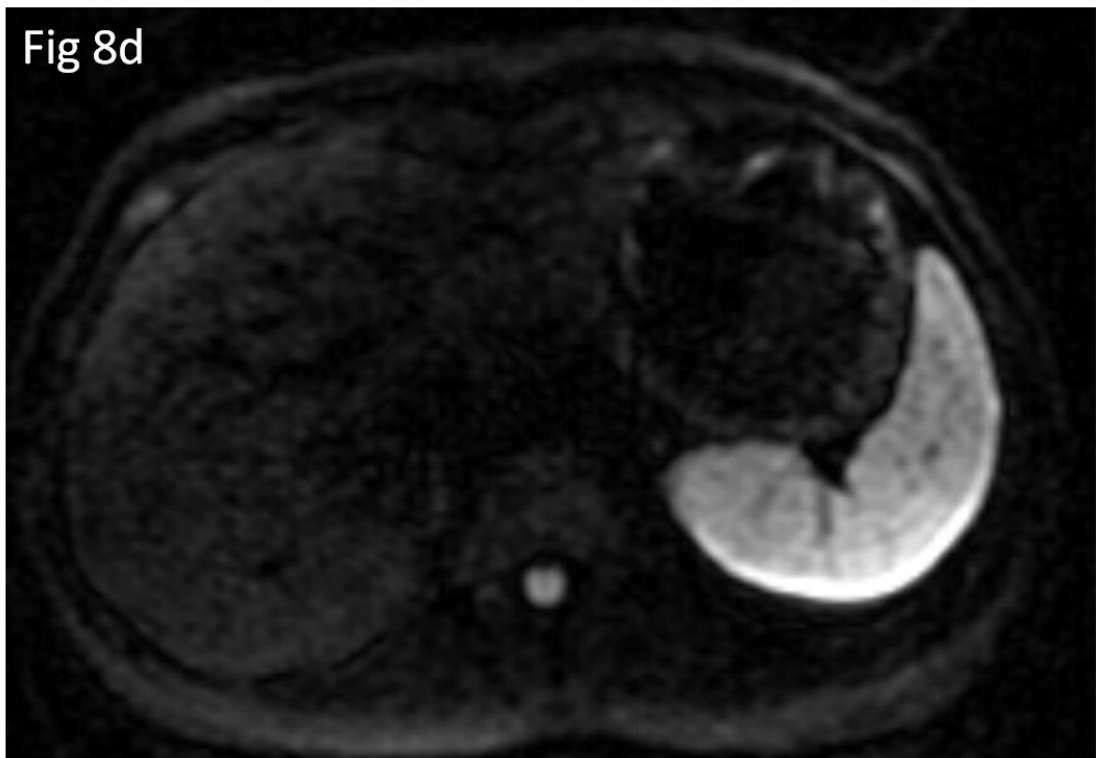

Supplement: S8 Fig — Both readers judged that the liver signal intensity was markedly reduced and the noise was significantly increased with increased B values, especially b-value = 800s/mm2, it was difficult to discriminate the boundry of vascular structures in left liver. (PDF) [file pone.0135568.s008.pdf]

**Fig 9. A patient with right liver lobe primary liver cancer.**

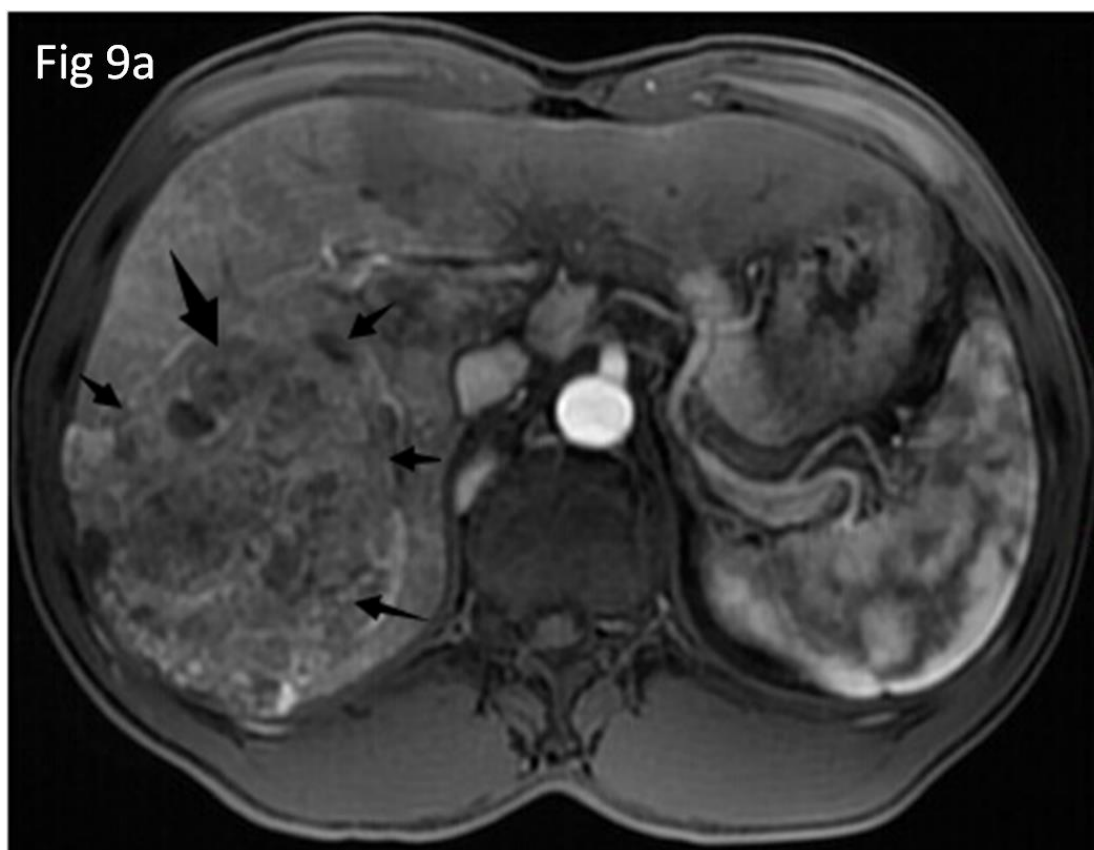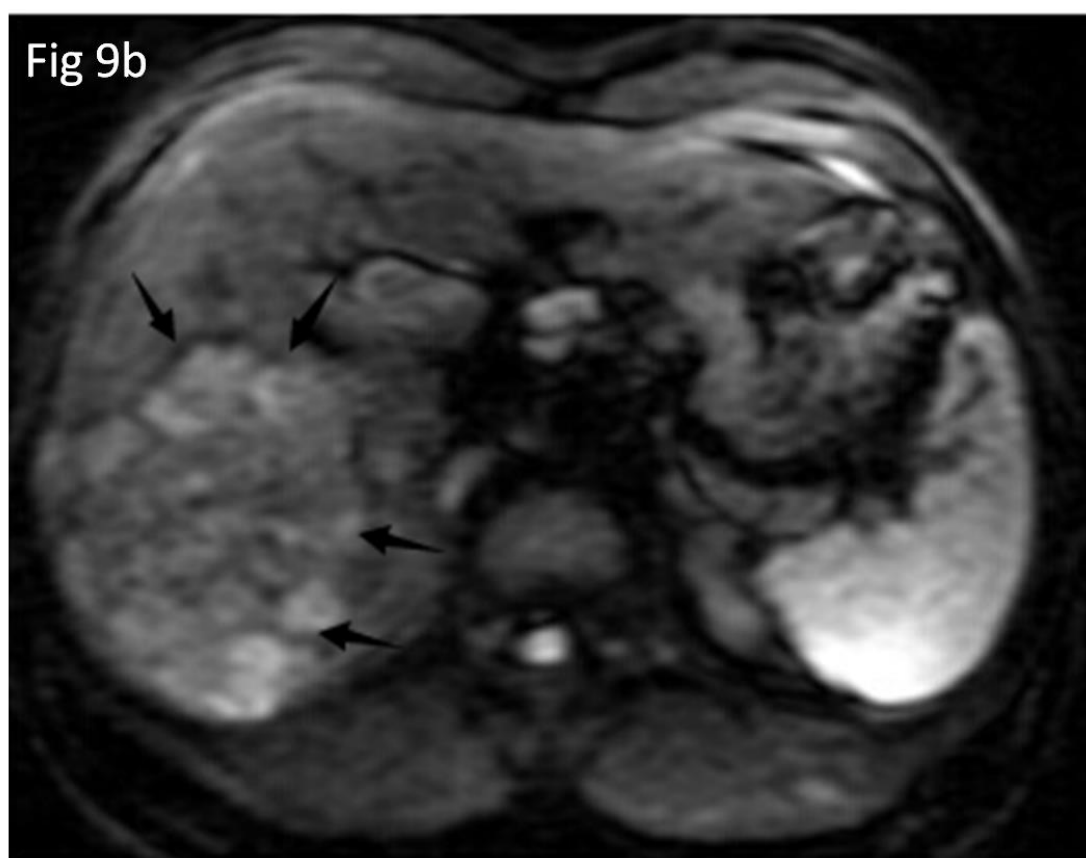

Fig 9c

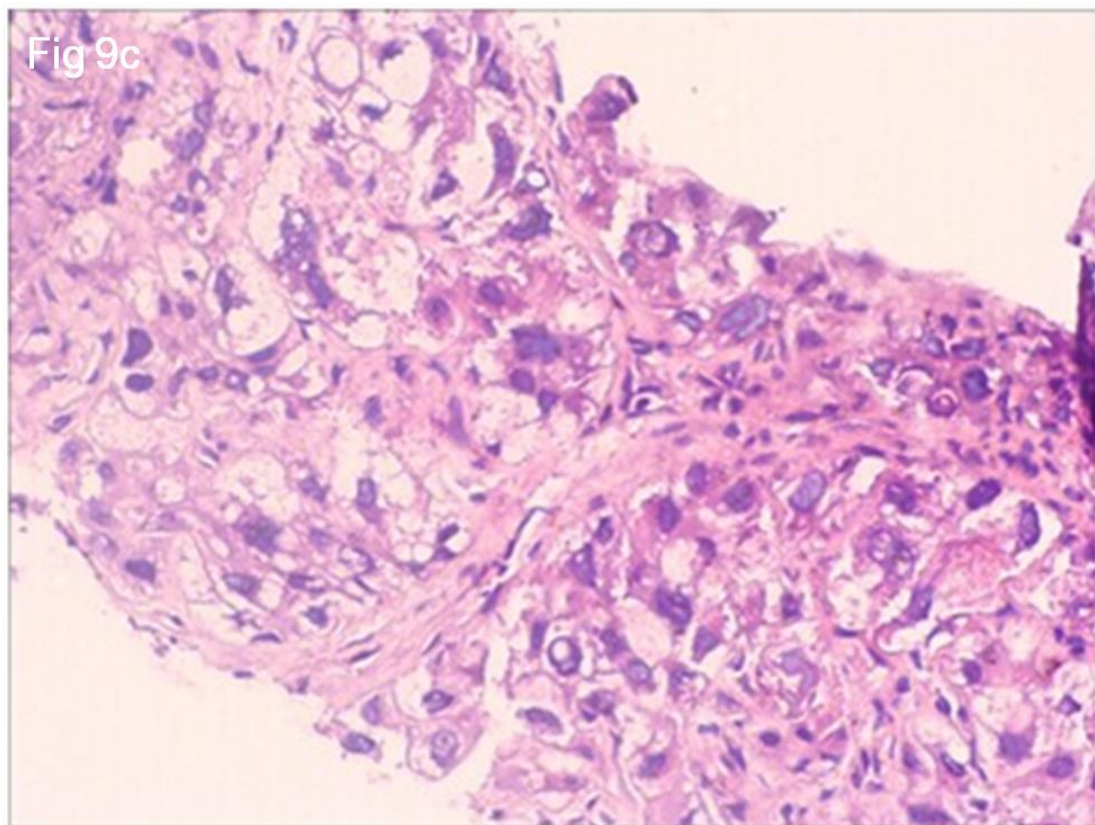

Supplement: S9 Fig — The tumor appeared hyperintense on DTI image (a). On dynamic gadolinium-enhanced imaging it showed markedly inhomogeneous enhancement at arterial phase (b). Surgical histopathologic diagnosis (c) confirmed it HCC. The tumor had an ADC value of 1.27×10^−3^mm2/s and a FA value of 0.33, respectively. (PDF) [file pone.0135568.s009.pdf]
