# Supplementary material for: Preliminary Study of MR Diffusion Tensor Imaging of the Liver for the Diagnosis of Hepatocellular Carcinoma
Source: PLoS One. 2015 Aug 28;10(8):e0135568. doi: 10.1371/journal.pone.0135568 (PMC4552840; doi:10.1371/journal.pone.0135568)

Fig 3. The main effect of b-values and NED on qualitative scores of liver DTI (a) and the interaction between b-values and NED on qualitative scores of liver DTI (b).

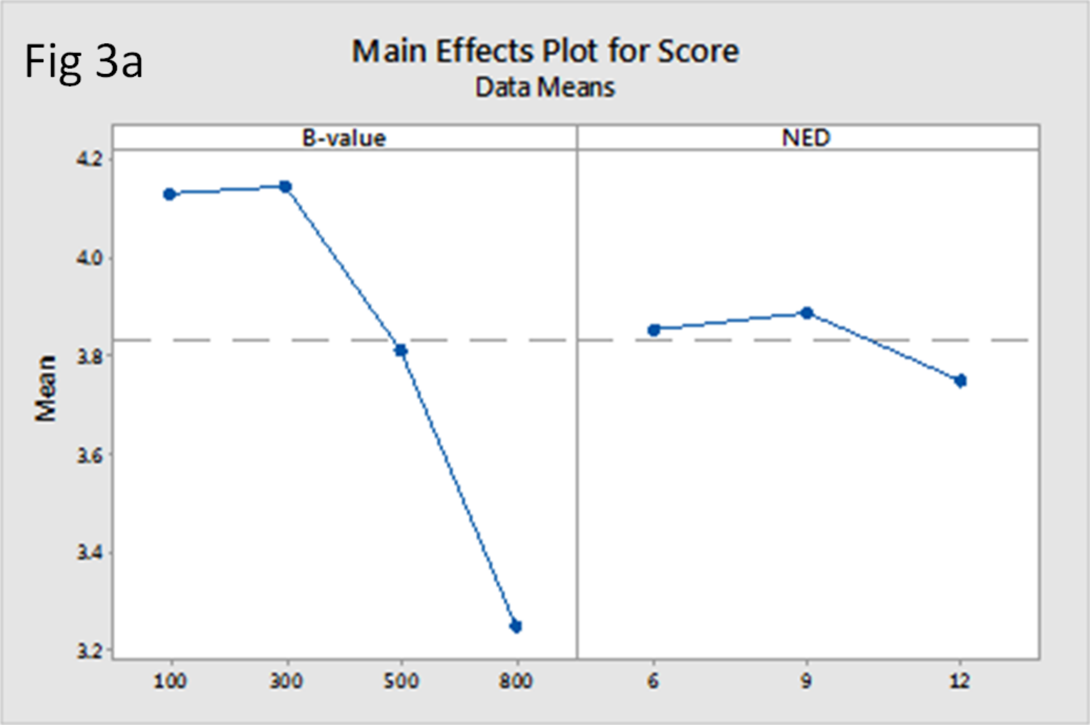

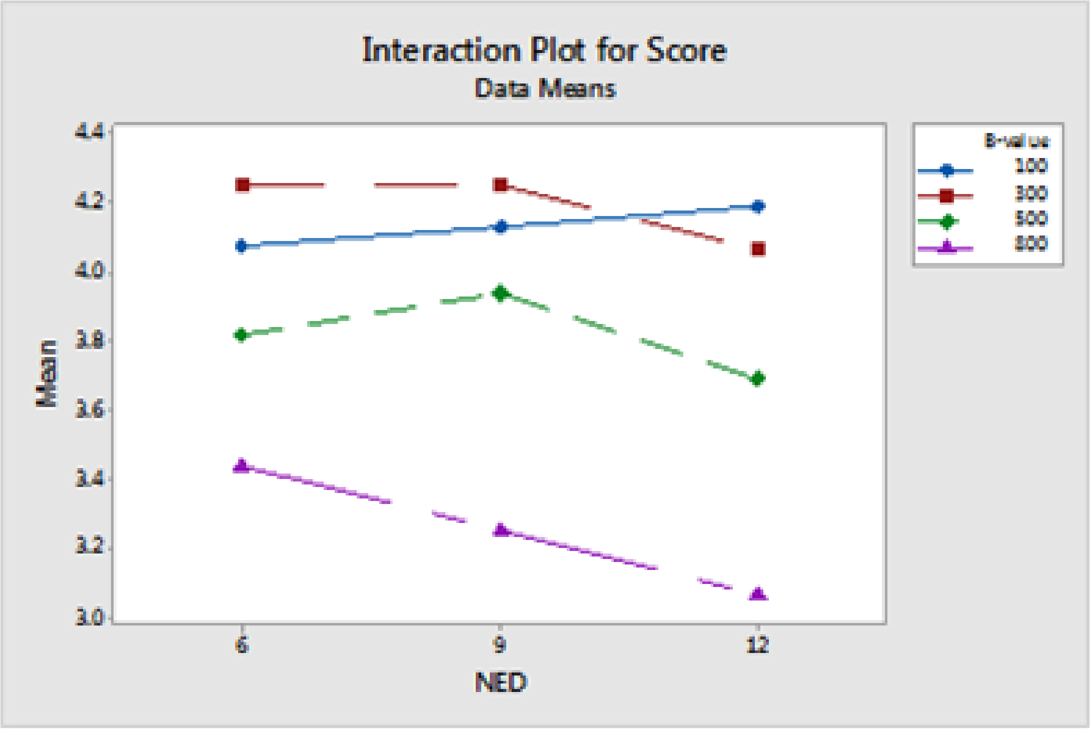

Supplement: S3 Fig — The main effect of b-values and NED on qualitative scores of liver DTI (a) showed that the main effect of NED on qualitative scores of liver DTI was not significant, but the scores of the image quality of liver reduced with increased b-values. The interaction between b-values and NED on qualitative scores of liver DTI (b) showed that choosing b-value = 100, 300 s/mm2 with NED = 9 could gain the higher score. (PDF) [file pone.0135568.s003.pdf]
