# Supplementary material for: Preliminary Study of MR Diffusion Tensor Imaging of the Liver for the Diagnosis of Hepatocellular Carcinoma
Source: PLoS One. 2015 Aug 28;10(8):e0135568. doi: 10.1371/journal.pone.0135568 (PMC4552840; doi:10.1371/journal.pone.0135568)

Fig 4. The main effect of b-values and NED on liver SNR (a) and the interaction between b-values and NED on liver SNR(b).

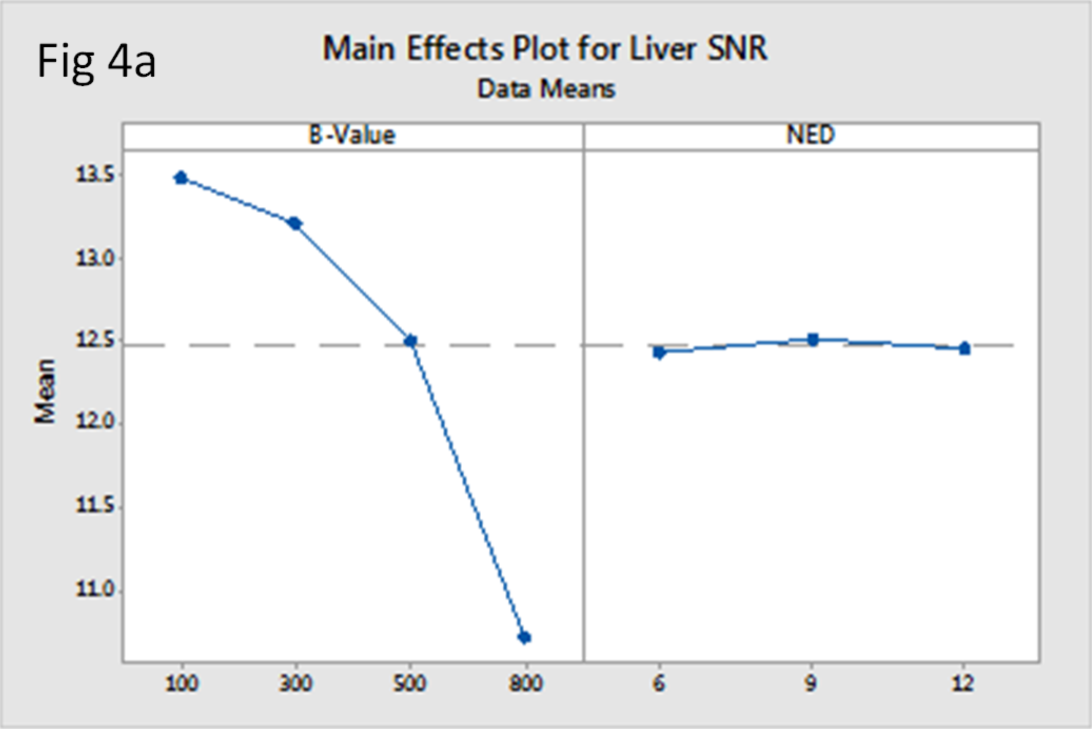

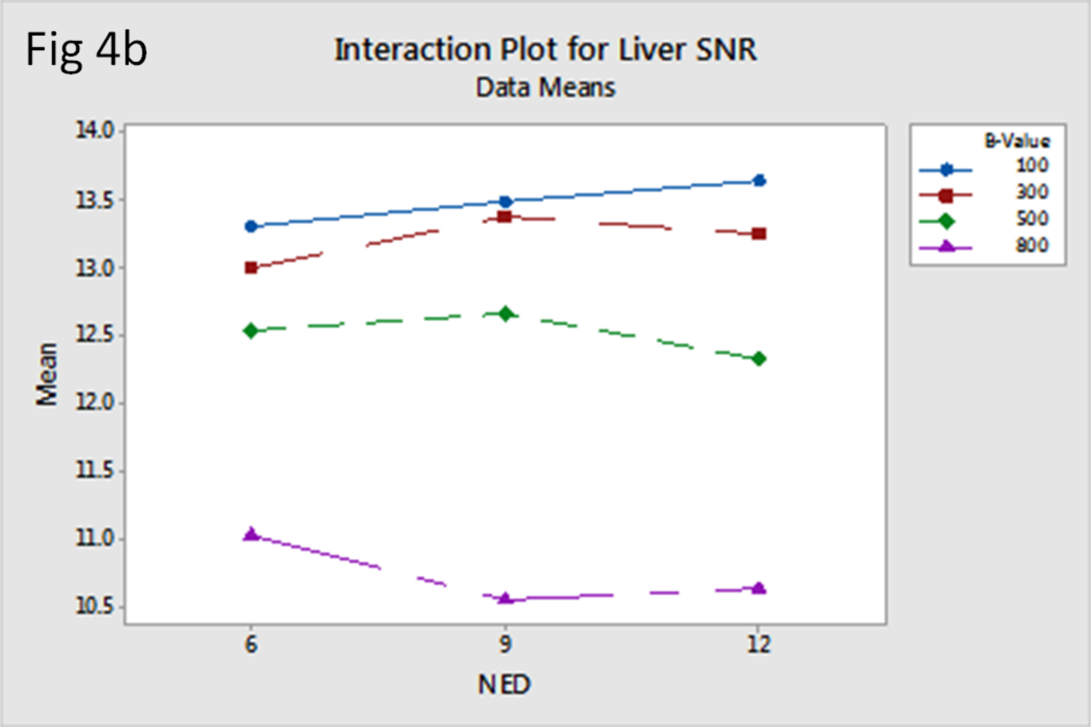

Supplement: S4 Fig — The main effect of b-values and NED on liver SNR (a) showed that the liver SNR reduced with increased b-values, but the differences had no significant with increased NED. The interaction between b-values and NED on liver SNR(b) showed that selected NED = 9 and b‑values range between 100 and 300s/mm2 lead to higher SNR of the liver DTI. (PDF) [file pone.0135568.s004.pdf]
