# Supplementary material for: Preliminary Study of MR Diffusion Tensor Imaging of the Liver for the Diagnosis of Hepatocellular Carcinoma
Source: PLoS One. 2015 Aug 28;10(8):e0135568. doi: 10.1371/journal.pone.0135568 (PMC4552840; doi:10.1371/journal.pone.0135568)
Supplement: S1 Table — (PDF) [file pone.0135568.s011.pdf]

Table 1.Comparison of the Five-Point-Scale Qualitative Scores of liver DTI with different B values and NED Effects.

B-value<br>NED	100 (s/mm2)	300 (s/mm2)	500 (s/mm2)	800 (s/mm2)	***F*(B)**	***P*(B)**
**6**	4.07(0.19)	4.15(0.48)	3.79(0.27)	3.50(0.41)		
**9**	4.13(0.44)	4.12(0.23)	3.94(0.32)	4.25(0.46)	**25.36**	**0.00***
**12**	4.19(0.26)	4.19(0.45)	3.75(0.27)	3.00(0.80)		
***F*(NED)**		**1.23**				
***P*(NED)**		**0.29**				

Note: The data are the mean image quality (standard deviation), Significant differences (P<0.05) are indicated with *. *F*(B) and *P*(B) represent the main effect of b-values on qualitative scores of liver DTI, *F*(NED) and *P*(NED) represent the main effect of NED on qualitative scores of liver DTI.
