# Supplementary material for: Preliminary Study of MR Diffusion Tensor Imaging of the Liver for the Diagnosis of Hepatocellular Carcinoma
Source: PLoS One. 2015 Aug 28;10(8):e0135568. doi: 10.1371/journal.pone.0135568 (PMC4552840; doi:10.1371/journal.pone.0135568)
Supplement: S2 Table — (PDF) [file pone.0135568.s012.pdf]

Table 2. Effects of b‐values and NED on liver SNR.

B-value<br>NED	100<br>(s/mm2)	300<br>(s/mm2)	500<br>(s/mm2)	800<br>(s/mm2)	***F*(B)**	***P*(B)**
**6**	13.30(3.37)	12.99(3.24)	12.54(1.82)	11.02(3.15)		
**9**	13.48(2.75)	13.37(4.18)	12.66(3.43)	10.55(1.60)	**3.21**	**0.03***
**12**	13.65(3.54)	13.25(3.46)	12.32(2.76)	10.62(3.22)		
***F*(NED)**		**0.73**				
***P*(NED)**		**0.48**				

Note: The data are the mean value (standard deviation). Significant differences ($P<0.05$) are indicated with *. *F*(B) and *P*(B) represent the main effect of b-values on SNR of liver DTI, *F*(NED) and *P*(NED) represent the main effect of NED on SNR of liver DTI.
