# Supplementary material for: Preliminary Study of MR Diffusion Tensor Imaging of the Liver for the Diagnosis of Hepatocellular Carcinoma
Source: PLoS One. 2015 Aug 28;10(8):e0135568. doi: 10.1371/journal.pone.0135568 (PMC4552840; doi:10.1371/journal.pone.0135568)
Supplement: S3 Table — (PDF) [file pone.0135568.s013.pdf]

Table 3. Effects of b‑values and NED on liver ADC.

B-value<br>NED	100 (s/mm2)	300 (s/mm2)	500 (s/mm2)	800 (s/mm2)	***F*(B)**	***P*(B)**
**6**	2.83(0.27)	1.73(0.32)	1.51(0.11)	1.40(0.12)		
**9**	3.05(0.80)	1.70(0.26)	1.54(0.20)	1.33(0.14)	**90.17**	**0.00***
**12**	2.65(0.52)	1.74(0.23)	1.46(0.17)	1.38(0.16)		
***F*(NED)**		**0.29**				
***P*(NED)**		**0.75**				

Note: The data are the mean value (standard deviation). The ADC values were equal to mean value×10−3mm^2/s. Significant differences (P<0.05) are indicated with *. . *F*(B) and *P*(B) represent the main effect of b-values on ADC of liver DTI, *F*(NED) and *P*(NED) represent the main effect of NED on ADC of liver DTI.
