# Supplementary material for: Preliminary Study of MR Diffusion Tensor Imaging of the Liver for the Diagnosis of Hepatocellular Carcinoma
Source: PLoS One. 2015 Aug 28;10(8):e0135568. doi: 10.1371/journal.pone.0135568 (PMC4552840; doi:10.1371/journal.pone.0135568)
Supplement: S4 Table — (PDF) [file pone.0135568.s014.pdf]

Table 4　Effects of b‑values and NED on liver FA

B-value<br>NED	100 (s/mm2)	300 (s/mm2)	500 (s/mm2)	800 (s/mm2)	*F*(B)	*P*(B)
**6**	0.59(0.14)	0.38(0.05)	0.43(0.09)	0.42(0.06)	**20.36**	**0.00***
**9**	0.53(0.11)	0.37(0.07)	0.38(0.09)	0.34(0.04)		
**12**	0.56(0.19)	0.36(0.10)	0.29(0.06)	0.33(0.05)		
***F*(NED)**		**3.14**				
***P*(NED)**		**0.05**				

Note: The data are the mean value (standard deviation). Significant differences (P<0.05) are indicated with *.
