# Supplementary material for: Preliminary Study of MR Diffusion Tensor Imaging of the Liver for the Diagnosis of Hepatocellular Carcinoma
Source: PLoS One. 2015 Aug 28;10(8):e0135568. doi: 10.1371/journal.pone.0135568 (PMC4552840; doi:10.1371/journal.pone.0135568)
Supplement: S5 Table — (PDF) [file pone.0135568.s015.pdf]

Table 5. Comparison of the FA and ADC value between HCC lesions and normal liver.

**Parameter**	**HCC lesions**	**Normal liver**	**P Value**
ADC value	1.30 (0.34)	1.52 (0.27)	**0.013***
FA value	0.42(0.11)	0.32 (0.10)	**0.004***

Note: The data are the mean ADC and FA ((standard deviation). The ADC values were equal to mean value×10−3mm^2/s. Significant differences ($P<0.05$) are indicated with *.
